# Identifying important microbial and genomic biomarkers for differentiating right- versus left-sided colorectal cancer using random forest models

**DOI:** 10.1186/s12885-023-10848-9

**Published:** 2023-07-11

**Authors:** Tyler Kolisnik, Arielle Kae Sulit, Sebastian Schmeier, Frank Frizelle, Rachel Purcell, Adam Smith, Olin Silander

**Affiliations:** 1 School of Natural Sciences, Massey University, Auckland, New Zealand; 2 Canada’s Michael Smith Genome Sciences Centre, BC Cancer, Vancouver, BC Canada; 3 Department of Surgery, University of Otago, Christchurch, New Zealand; 4 School of Mathematical and Computational Sciences, Massey University, Auckland, New Zealand

**Keywords:** Colorectal Cancer, Left-sided colon cancer, Right-sided colon cancer, Machine learning, Microbiome

## Abstract

**Background:**

Colorectal cancer (CRC) is a heterogeneous disease, with subtypes that have different clinical behaviours and subsequent prognoses. There is a growing body of evidence suggesting that right-sided colorectal cancer (RCC) and left-sided colorectal cancer (LCC) also differ in treatment success and patient outcomes. Biomarkers that differentiate between RCC and LCC are not well-established. Here, we apply random forest (RF) machine learning methods to identify genomic or microbial biomarkers that differentiate RCC and LCC.

**Methods:**

RNA-seq expression data for 58,677 coding and non-coding human genes and count data for 28,557 human unmapped reads were obtained from 308 patient CRC tumour samples. We created three RF models for datasets of human genes-only, microbes-only, and genes-and-microbes combined. We used a permutation test to identify features of significant importance. Finally, we used differential expression (DE) and paired Wilcoxon-rank sum tests to associate features with a particular side.

**Results:**

RF model accuracy scores were 90%, 70%, and 87% with area under curve (AUC) of 0.9, 0.76, and 0.89 for the human genomic, microbial, and combined feature sets, respectively. 15 features were identified as significant in the model of genes-only, 54 microbes in the model of microbes-only, and 28 genes and 18 microbes in the model with genes-and-microbes combined. *PRAC1* expression was the most important feature for differentiating RCC and LCC in the genes-only model, with *HOXB13*, *SPAG16*, *HOXC4*, and *RNLS* also playing a role. *Ruminococcus gnavus* and *Clostridium acetireducens* were the most important in the microbial-only model. *MYOM3*, *HOXC4*, *Coprococcus eutactus*, *PRAC1*, lncRNA AC012531.25, *Ruminococcus gnavus*, *RNLS*, *HOXC6*, *SPAG16* and *Fusobacterium nucleatum* were most important in the combined model.

**Conclusions:**

Many of the identified genes and microbes among all models have previously established associations with CRC. However, the ability of RF models to account for inter-feature relationships within the underlying decision trees may yield a more sensitive and biologically interconnected set of genomic and microbial biomarkers.

**Supplementary Information:**

The online version contains supplementary material available at 10.1186/s12885-023-10848-9.

## Background

Despite being part of the same organ, colorectal cancer tumours can have different pathogenicity, histology and patient outcomes depending on subtype [1] and which side of the splenic flexure they occur [2]. Left-sided colorectal cancer (LCC, or distal colorectal cancer) affects the rectum, sigmoid colon, descending colon, and distal one-third of the transverse colon. It is generally more common in men, diagnosed at an earlier stage, more responsive to treatment, and patients exhibit a higher rate of survival [3]. Right-sided colorectal cancer (RCC, or proximal colorectal cancer) affects the proximal two-thirds of the transverse colon, ascending colon, and caecum [3]. It is generally more common in women, less responsive to existing treatments, and has poorer outcomes [2]. Numerous studies have reported vast differences between LCC and RCC in terms of diagnostics, prognostics, histology, epidemiology, pathology, treatment response, and survival [4–6]. Among other things, these differences suggest that LCC and RCC should be distinguished when developing new treatment regimens and therapeutic drugs [7, 8].

Gut microbiota has been shown to play an influential role in CRC carcinogenesis and progression. However, the mechanisms by which this occurs largely remains unknown [9]. In addition to cancer progression, it has also been postulated that the gut microbiome may affect gene expression and downstream patient treatment responses [10]. To test these hypotheses, there is a need for studies that explore the influence of the gut microbiome on the genomic expression inside colorectal cancer tumor cells.

Machine learning (ML) methods are frequently applied for classification in tasks that rely on high-dimensional genomic data. Here, to query the relationships between the expression of genomic features in CRC and microbial content, we use Random Forest (RF) classification [11]. We selected RF as it can account for interactions and correlations among large numbers of features [12]. Furthermore, RF models do not require normalization or scaling, which makes it possible to combine completely different types of data, for example, microbial count data and RNA-seq datasets.

Here, we explore the ability of RF models to predict CRC sidedness using three different datasets: human genomic feature expression level (RNA-seq), microbial count data (from unmapped human reads), and a combined genomic feature and microbial count dataset. We subsequently use differential expression (DE) analysis on the most important features of the RF model (i.e., biomarkers) to find differential genomic and microbial features and relationships between RCC and LCC. Finally, we discuss the possible biological mechanisms driving differences in these biomarkers.

## Methods & materials

### Patients, samples and processing

308 colorectal cancer tumour samples were obtained from patients via surgical resection (partial colectomy). Patients with inherited CRC and those who had received preoperative chemotherapy or radiotherapy were excluded. Patients were over the age of 18 and gave written informed consent. Tumour tissue was obtained between January 2002 and January 2016, with a median tumour tissue date of August 2006. The study was approved by the University of Otago, New Zealand, Human Research Ethics Committee (approval number: H16/037). Patient and clinical data, including anatomical site of tumour, in addition to genomic and microbial data profiles were available for all patients. Samples were snap frozen in liquid nitrogen at time of surgery and stored at -80 °C and transitioned for RNA Extraction using RNAlater®-ICE. RNA was then extracted using the QIAGEN RNAEasy mini kit and sequenced using Illumina HiSeq machines (2 × 125 bp PE v4 sequencing). The samples were machine-randomized to limit any machine-specific noise or calibration bias. Raw Sequence Reads are available at SRA Accession: PRJNA788974.

Sequence reads were first mapped to the human genome (GRCh38) using STAR (v2.73a). The remaining unmapped reads were classified using Kaiju (v1.6.2) to obtain microbial abundances [13, 14]. Raw genomic reads were TPM (transcripts per kilobase million) normalised prior to data analysis to remove gene length and sequencing depth biases. Microbial abundances were CPM (Counts per million) normalized.

### Random forest model generation

The RF models were built on the following training datasets: the first contained 58,677 TPM normalized genomic features, the second contained CPM normalized microbial counts for 28,557 taxa, and the third contained a combination of both. A separate validation cohort of 30 samples (15 RCC, 15 LCC) was held out from model generation, leaving 278 patient samples for model development. Genomic and microbial data was available for all 308 patients.

The RF models were parametrized in parallel on high-powered cluster computing nodes with 8,136 cores in 226 × Broadwell nodes, and a total system memory of 31 TB.

**Table 1:** Patient Demographics & Cancer Characteristics

Characteristic	Value
**Patients enrolled - no (%)**	308 (100)
**Median Age - year (range)**	73 (28–91)
**Sex**	
Female - no (%)	163 (53)
Male - no (%)	145 (47)
**Cancer Anatomical Side**	
Left - no (%)	172 (56)
Right - no (%)	136 (44)
**Metastasis**	
Positive - no (%)	70 (23)
Negative - no (%)	238 (77)
**Ethnicity (Self-Reported)**	
European - no (%)	296 (96)
Māori - no (%)	9 (3)
Asian - no (%)	3 (1)
**Cancer Stage**	
T1 - no (%)	53 (17)
T2 - no (%)	128 (42)
T3 - no (%)	105 (34)
T4 - no (%)	22 (7)
**Nodal Status**	
Positive - no (%)	185 (60)
Negative - no (%)	123 (40)

RF models were generated using the Python-based scikit-learn random forest module [15, 16]. Model hyperparameters were optimized independently for all three training datasets using a grid search with 5-fold cross validation (scikit-learn package GridSearchCV) [15]. To narrow down suitable hyperparameter sets, the influence of 8 hyperparameters on F1 scores (the weighted average of precision and recall) were each independently observed across a typical range of values for each, while holding the other hyperparameters to their default values (Supplementary Figs. 1–3). GridSearchCV with 5-fold cross validation was then used on the smaller set of hyperparameter combinations on the training datasets. A final set of model parameters was selected for each dataset based on highest performing receiver-operator characteristic area-under the curve score (AUROC score), accuracy (total correctly classified cases), and F1 score. For cases in which the performance scores were identical, the model with the fewest features was selected.

Each model was trained using the finalized set of parameters using a 75% train, 25% test split on the dataset of tumor samples from the 278 different patients. The model metrics of accuracy, out-of-bag score, f1 score, ROC AUC score, recall, and precision for each of the three models are reported in Table 2. Overfitting was assessed by comparing model accuracy with out-of-bag score (number of correct predictions in the out-of-bag sample) and accuracy of the validation cohort. If the model accuracy differed from the out-of-bag score by 0.1 or more, we inferred that there was a strong likelihood overfitting had occurred. A threshold analysis was also performed for each model, but we found that all optimised thresholds were within 10% of the default value, so we used the default threshold value (0.5). The model was then validated on our validation cohort of 30 samples, this is sometimes referred to as the testing set, and is independent from the testing data used in model training. ROC curves were generated for all three models using the Python package seaborn [17].

### Feature importance and retention

The feature importance scores (Gini impurity values) were extracted from each of our three RF models. Given that the models have large numbers of features (greater than 50), it is prudent to perform feature reduction such that only features with high importance (weight) and a high degree of statistical evidence are retained. Using the R package Rf2pval [18], we implemented a rank-based permutation approach to obtain distributions of feature-importance scores at each rank under a null hypothesis where none of the features are associated with the target variable, and assign p-values to the features. We generated 100 randomized datasets in which the target variable (‘side’) was permuted, retrained the RF models on each, and obtained feature importance scores and scoring metrics for each permuted model. We retained only features with p-values less than 0.05 (Fig. 2a-c). A threshold for feature reduction was identified using the overlap of feature importance scores from the true model with the mean of the permuted feature importance scores (Tables 1, 2 and 3). Family-wise error rate via resampling was used for measuring the probability of making one or more false discoveries during multiple-testing and was calculated using the Rf2pval package for all three models to be FWER < 0.05, or less than 5% chance of our features listed above our threshold being incorrectly identified.

**Table 2:** Random Forest Model Results

Scoring Metric	Model		
Testing set (5-Fold CV)	Genes-Only	Microbes-Only	Genes-and-Microbes
Accuracy	0.94	0.76	0.8
Out-of-Bag Score	0.73	0.74	0.74
F1 Score	0.95	0.79	0.84
ROC AUC Score	0.94	0.75	0.78
Recall Score	0.95	0.8	0.93
Precision Score	0.95	0.78	0.77
**Validation Set (30 held-out samples)**			
Accuracy	0.9	0.7	0.87
F1 Score	0.9	0.76	0.88
ROC AUC Score	0.9	0.76	0.89
Recall Score	0.93	0.64	0.79
Precision Score	0.87	0.93	1

**Table 3:** Top ranking features from the RF model trained on the genes-only dataset (Left). Top ranking features with p-values less than 0.05 and their importance scores discovered by our genes model (Left). Side-paired differential expression (fold change) analysis results of TPM values for the same features (Right) Wilcoxon-rank sum test was used to calculate p-values and FDR (Benjamini & Hochberg)

Model Feature Importance Metrics					Differential Expression			
Rank	Ensemble ID_Gene ID	Importance Score	Log Importance Score	p-value	Log2 FC	p-value	FDR	Greater Expr. Side
1	ENSG00000159182_PRAC1	0.12	-2.16	0	-2.86	5.08E-21	3.10E-19	Left
2	ENSG00000159184_HOXB13	0.07	-2.7	0	-1.78	1.78E-11	2.18E-10	Left
3	ENSG00000144451_SPAG16	0.05	-3.04	0	-0.64	9.00E-09	4.99E-08	Left
4	ENSG00000198353_HOXC4	0.05	-3.08	0	1.88	2.11E-15	6.43E-14	Right
5	ENSG00000184719_RNLS	0.03	-3.36	0	-0.89	2.85E-10	2.48E-09	Left
6	ENSG00000145649_GZMA	0.03	-3.42	0	1.54	4.75E-07	1.93E-06	Right
7	ENSG00000197757_HOXC6	0.02	-3.84	0	1.28	5.02E-12	1.02E-10	Right
8	ENSG00000162409_PRKAA2	0.02	-3.87	0	-1.2	3.90E-10	2.97E-09	Left
9	ENSG00000037965_HOXC8	0.02	-3.87	0	1.32	7.90E-12	1.20E-10	Right
10	ENSG00000147457_CHMP7	0.02	-3.92	0	0.35	8.44E-06	2.71E-05	Right
11	ENSG00000165548_TMEM63C	0.02	-3.99	0	-0.83	5.31E-05	0.000147	Left
12	ENSG00000203880_PCMTD2	0.02	-4.01	0	-0.43	5.16E-08	2.62E-07	Left
13	ENSG00000119397_CNTRL	0.02	-4.08	0.01	0.27	1.84E-06	7.00E-06	Right
14	ENSG00000103485_QPRT	0.02	-4.09	0.01	-1.01	2.20E-10	2.24E-09	Left
15	ENSG00000170677_SOCS6	0.02	-4.13	0.03	0.39	4.14E-06	1.40E-05	Right

### Differential expression, feature side-assignment and heatmap generation

DE analysis was performed on each of the feature lists from the three models using edgeR [18]. Wilcoxon-rank sum tests were used to calculate p-values to test for DE of each model’s features. Heatmaps were generated for assessing feature clustering compared with the clinical labels of cancer stage, metastasis, subtype, gender site and side using the function heatmap.2 in the R package gplots [19]. The heatmap implements row-scaled z-scores of the transcripts per kilobase million (TPM) read counts, with hierarchical clustering using Pearson distance correlation, and average-linkage distance.

## Results

### Random forest model performance

**Fig. 1:**
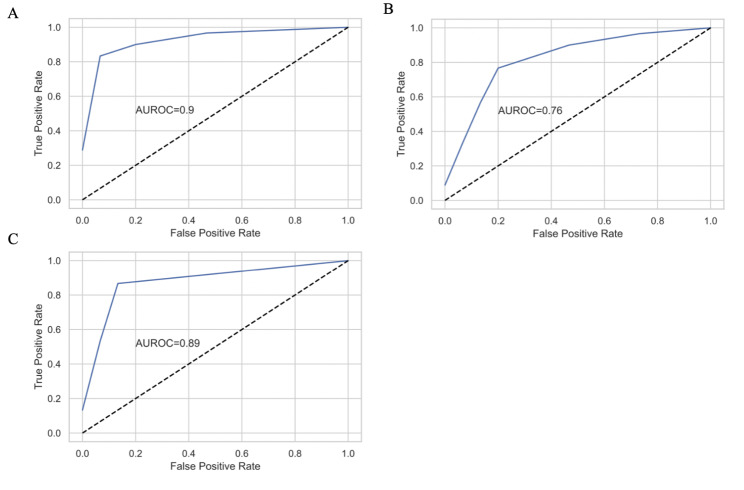
Receiver Operating Characteristic Curves (ROC) as calculated on the held-out validation set. **a** ROC curve of the genes-only model. **b** ROC curve of the microbial-only model. **c** ROC curve of the genes-and-microbes model

**Fig. 2:**
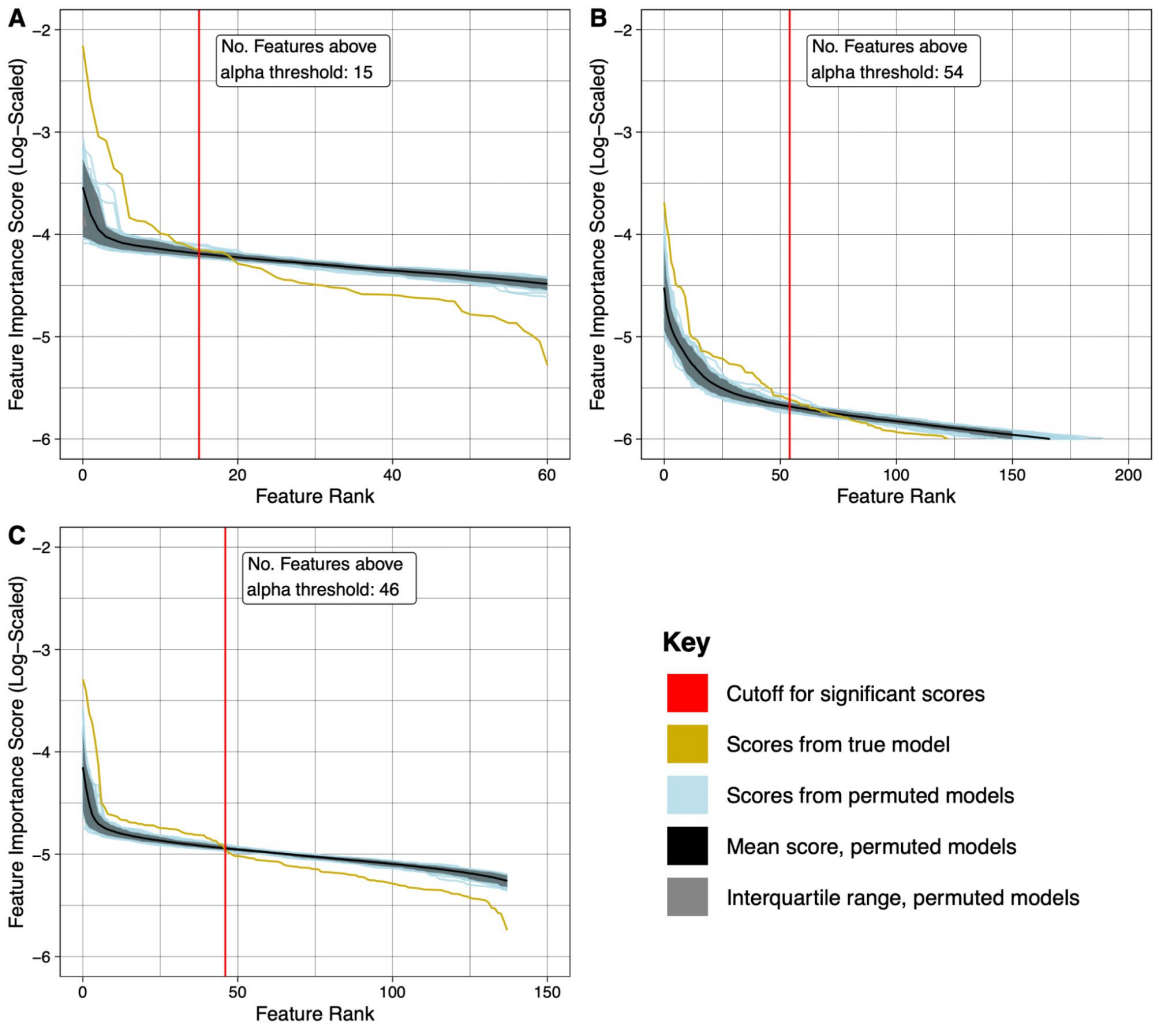
Feature importance plots showing rank-based feature importance scores of the permuted data and the scores of the real (unpermuted) data. The cutoff for features reported as significant was determined based on an alpha threshold of 0.05, and are to the left of the vertical red line. **a** genes-only model. **b** microbes-only model. **c** shows the genes-and-microbes model

We found that the random forest models from all three datasets clearly differentiated between LCC and RCC. Model accuracy on the validation sets ranged from 0.7 to 0.9, with genomic features having an accuracy of 0.94 and 0.9 on the training and validations sets, respectively microbial counts having an accuracy of 0.76 and 0.7, and genomic features with microbial counts having an accuracy of 0.8 and 0.87 (Fig. 1). Out-of-bag scores were 0.73, 0.74 and 0.74 for the three datasets. The strongest predictors between the LCC and RCC were genomic features, although classifications based on microbial count differences were also consistent. We found 15 statistically significant features in the genes-only model, 54 in the microbes-only model, and 46 in the genes-and-microbes model (Fig. 2A-C; Tables 3, 4 and 5).

**Table 4:** As per Table 3, with microbes-only model data. Top ranking features with p-values less than 0.05 and their importance scores discovered by our microbes-only model (Left). Side-paired differential expression (fold change) analysis results of CPM values for the same features (Right) Wilcoxon-rank sum test was used to calculate p-values and FDR (Benjamini & Hochberg)

Model Feature Importance Metrics					Differential Expression			
Rank	Tax ID_Name	Importance Score	Log Importance Score	p-value	Log2 FC	p-value	FDR	Greater Expr. Side
1	33038_[Ruminococcus] gnavus	0.025	-3.69	0	2.07E + 00	1.31E-15	3.54E-14	Right
2	76489_Clostridium acetireducens	0.020	-3.91	0	1.88E + 00	1.69E-13	1.52E-12	Right
3	1701326_uncultured bacterium 5G4	0.018	-4.04	0	1.73E + 00	6.55E-11	2.52E-10	Right
4	397291_Lachnospiraceae bacterium A4	0.014	-4.27	0	2.30E + 00	5.29E-16	2.85E-14	Right
5	2293240_Ruminococcus sp. TF10-6	0.013	-4.34	0	2.41E + 00	2.86E-15	5.14E-14	Right
6	239935_Akkermansia muciniphila	0.011	-4.51	0	-5.30E-01	2.27E-05	3.96E-05	Left
7	1531_[Clostridium] clostridioforme	0.011	-4.55	0	1.47E + 00	1.73E-12	1.03E-11	Right
8	936381_Selenomonas sp. CM52	0.010	-4.61	0	4.03E + 00	8.68E-11	3.13E-10	Right
9	46228_Ruminococcus lactaris	0.009	-4.76	0	1.86E + 00	2.96E-12	1.45E-11	Right
10	43064_Trichococcus pasteurii	0.007	-4.97	0	2.18E + 00	1.67E-08	4.10E-08	Right
11	1262831_Clostridium sp. CAG:678	0.007	-5.01	0	1.3	0.0322	0.0464	Right
12	1824_Nocardia asteroides	0.007	-5.02	0	-0.19	0.00112	0.00172	Left
13	208479_[Clostridium] bolteae	0.007	-5.03	0	1.76E + 00	2.18E-13	1.68E-12	Right
14	2026799_Verrucomicrobia bacterium	0.006	-5.14	0.01	-0.17	2.02E-06	3.76E-06	Left
15	1262706_Azospirillum sp. CAG:260	0.006	-5.14	0	1.15	1.86E-09	5.57E-09	Right

### Significant model features

Of the 15 significant features in our gene-only dataset, (Fig. 2A; Table 3) the highest importance score was for the Prostate Cancer Susceptibility Candidate 1 (*PRAC1*) gene, which has higher expression in LCC. Other significantly important features included those in the *HOX* family of genes, *HOXB13*, *HOXC4*, *HOXC6* and *HOXC8*.

Table 3. Top ranking features from the RF model trained on the genes-only dataset (Left). Top ranking features with p-values less than 0.05 and their importance scores discovered by our genes model (Left). Side-paired differential expression (fold change) analysis results of TPM values for the same features (Right) Wilcoxon-rank sum test was used to calculate p-values and FDR (Benjamini & Hochberg).

In the microbes-only dataset, 54 features were identified by Rf2pval as significantly important (Fig. 2B; Table 4). The taxon with the highest importance score was *Ruminococcus gnavus*, which shows higher counts in RCC (Table 4). *Clostridium acetireducens* ranked second and was more abundant in RCC.

**Table 5:** As per Tables 3 and 4, with genes-and-microbes model data. Top ranking features with p-values less than 0.05 and their importance scores discovered by our genes-and-microbes model (Left). Side-paired differential expression (fold change) analysis results of TPM and CPM values for the same features (Right) Wilcoxon-rank sum test was used to calculate p-values and FDR (Benjamini & Hochberg)

Model Feature Importance Metrics					Differential Expression			
Rank	ENSG ID_Gene/Tax ID_Name	Importance Score	Log Importance Score	p-value	Log2 FC	p-value	FDR	Associated Side
1	ENSG00000142661_MYOM3	0.037	-3.29	0	-0.62	1.36E-08	2.71E-08	Left
2	ENSG00000198353_HOXC4	0.033	-3.40	0	1.88	2.11E-15	3.23E-14	Right
3	33043_Coprococcus eutactus	0.027	-3.62	0	2.08	2.00E-14	1.54E-13	Right
4	ENSG00000159182_PRAC1	0.024	-3.72	0	-2.86	5.08E-21	2.34E-19	Left
5	ENSG00000260597_AC012531.25	0.020	-3.90	0	1.19	4.26E-12	2.17E-11	Right
6	33038_[Ruminococcus] gnavus	0.016	-4.13	0	2.07	1.31E-15	3.02E-14	Right
7	ENSG00000184719_RNLS	0.011	-4.50	0.01	-0.89	2.85E-10	9.37E-10	Left
8	ENSG00000197757_HOXC6	0.011	-4.54	0	1.28	5.02E-12	2.31E-11	Right
9	ENSG00000144451_SPAG16	0.010	-4.61	0	-0.64	9.00E-09	1.97E-08	Left
10	851_Fusobacterium nucleatum	0.010	-4.62	0	1.67	2.73E-06	3.49E-06	Right
11	ENSG00000273374_RP11-383I23.2	0.010	-4.62	0	-1.02	2.86E-08	5.47E-08	Left
12	446043_uncultured Lachnospira sp.	0.010	-4.63	0	1.46	5.97E-09	1.37E-08	Right
13	165179_Prevotella copri	0.009	-4.66	0	1.6	1.50E-09	3.85E-09	Right
14	154288_Turicibacter sanguinis	0.009	-4.67	0	1.9	5.00E-08	8.51E-08	Right
15	59620_uncultured Clostridium sp.	0.009	-4.67	0	1.09	6.86E-12	2.87E-11	Right

Table 4. As per Table 3, with microbes-only model data. Top ranking features with p-values less than 0.05 and their importance scores discovered by our microbes-only model (Left). Side-paired differential expression (fold change) analysis results of CPM values for the same features (Right) Wilcoxon-rank sum test was used to calculate p-values and FDR (Benjamini & Hochberg).

46 features were deemed significant in the genes-and-microbes model (Fig. 2C; Table 5): 28 genomic features and 18 microbes. Notable features include *MYOM3*, *HOXC4*, *Coprococcus eutatus*, *PRAC1*, lncRNA AC012531.3, *Ruminococcus gnavus*, *RNLS*, *HOXC6*, *SPAG16*, and *Fusobacterium nucleatum*.

Table 5. As per Tables 3 and 4, with genes-and-microbes model data. Top ranking features with p-values less than 0.05 and their importance scores discovered by our genes-and-microbes model (Left). Side-paired differential expression (fold change) analysis results of TPM and CPM values for the same features (Right) Wilcoxon-rank sum test was used to calculate p-values and FDR (Benjamini & Hochberg).

**Fig. 3:**
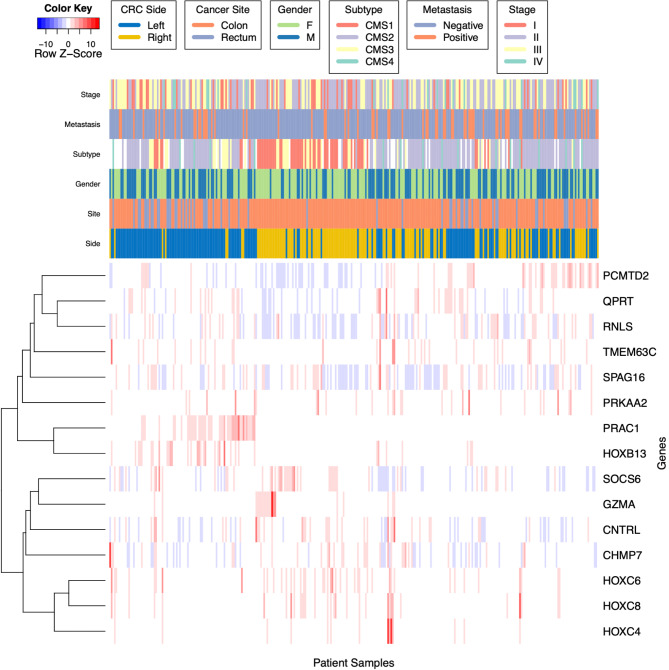
A heatmap of scaled gene expression values of the top-scoring genomic features discovered by the genes-only RF model and clinical characteristics. Hierarchical clustering of both genes and patients is via Pearson correlation, based on average linkage distance. The colors indicate row-scaled z-scores of TPM RNA-seq gene expression ratios

We used hierarchical clustering to ascertain connections between gene expression profiles and clinical characteristics and consensus subtyping scores (Fig. 3). As expected, of the six clinical characteristics that we considered (cancer stage, post-operative metastasis, consensus molecular subtype (CMS), gender, and site), side is most closely linked to the gene expression levels of our top genomic features. There is a clear cluster of left-sided CRC samples that show higher expression levels of *PRAC1* and *HOXB13* (left side of heatmap). There is also a subset of RCC that show higher expression of *HOXC4*, *HOXC6*, and *HOXC8* (middle of heatmap), although not all RCC exhibit this pattern. Heatmap for microbes-only model is shown in Supplementary Fig. 4, and the heatmap for genes-and-microbes model is shown in Supplementary Fig. 5.

In total, our models discovered 107 unique genomic and microbial features which played a significant role in the differentiating between CRC anatomical sides. Only six genomic features were common to both the genomic and genomic-plus-microbial models: *PRAC1*, *SPAG16*, *HOXC4*, *RNLS*, *HOXC6* and *PRKAA2*; and six microbes which were common to both our microbes-only, and genes-and-microbes models: *Ruminococcus gnavus, Ruminococcus sp. TF10-6, Selenomonas sp. CM52, Verrucomicrobia bacterium, Anaerostipes caccae* and *Turicibacter sanguinis*.

## Discussion

Many of the previous studies on RCC vs. LCC and gene expression have used the publicly available TCGA data. Here, we used a novel dataset of 308 patients, with microbial data from human unmapped reads, which adds to the growing body of evidence of the genomic and microbial differences between the sites [20, 21].

One difficulty in characterizing the roles of the microbiome and the genome in RCC vs. LCC is that there is genomic and microbial heterogeneity both between and within the two anatomical locations [22]. A primary reason for this heterogeneity is that the proximal and distal areas of the colon have different embryonic origins and physiological functions: the right-side of the colon is derived from the embryonic midgut and is involved in digestion, and the left side of the colon is derived from the embryonic hindgut and is involved primarily in the storage of fecal matter and water absorption. Despite these different functions, the microbial content is similar in these two parts of the colon because they are attached, and peristaltic movement allows stool matter to pass both forwards and backwards [23]. Numerous studies have shown a strong correlation between gut dysbiosis and CRC, but less is known about the microbial taxa that differentiate RCC and LCC and, perhaps, play a role in carcinogenesis [24]. *Fusobacterium*, *Prevotella*, *Clostridium, Akkermansia*, and *Ruminococcus* are among the most frequently reported bacteria in studies on CRC-related microbial dysbiosis [24, 25]. All were deemed significantly important microbial taxa in the RF models presented here.

All three RF models showed strong predictive accuracy. The microbes-only model showed the poorest predictive capability while the genes-only model was the highest performing. It is perhaps surprising that the combined model was not the most predictive. We postulate that this may be due to the fact that while microbes and genes may both affect CRC, microbial taxa are in fact indirect players, with effects that are reflected as altered genomic expression within the tumour, leading to cancer growth.

Finally, there were a number of highly important genes that differed between the genomic features only and combined RF models. One other point of interest is that there are some different top genes in the genes-only model when compared with the genes-and-microbes model. This may suggest that these genes and microbes act in consort and our genes-and-microbes RF model may have identified some underlying biological interactions.

### Patterns in RCC

The RF models showed that increased expression of the *HOX* family of genes was characteristic of RCC. Specifically, we observed an upregulation of *HOXC4*, *HOXC6*, *HOXC8*, and *HOX*-related lncRNA AC012531.3, and a downregulation of *HOXB13* (Tables 3 and 5). The *HOX* (homeobox) gene family is most well-known for guiding embryonic development [26]. *HOX* mutations that cause either increased or decreased expression have been associated with several types of cancer [27] as tumor suppressors and proto-oncogenes. However, their role in CRC is not well understood [27].

The top microbes identified by the microbes-only model include *Ruminococcus gnavus, Clostridium acetereducens*, *Lachnospiraceae*, and, *Ruminococcus sp. TF10-6* (Table 4). *R. gnavus* causes inflammation in Crohn’s disease models, and influences immunotherapy responses in CRC [28, 29]. *C. acetireducens* is an anaerobic bacterium that has no previously known associations to CRC. However, it is known to oxidize alanine to produce butyrate, and butyrate has been associated with CRC tumorigenesis [30]. *Lachnospiraceae* spp are also known to produce short-chain fatty acids which are known to have increased abundance in CRC patients [31]; *Ruminococcus sp. TF10-6*, also more abundant in RCC; and *Akkermansia muciniphila*, more abundant in LCC. There is some evidence that the largely uncharacterized lncRNA AC012531.3 which is located in one of the *HOX* gene loci, plays a role in colorectal cancer carcinogenesis [32].

For the genes-and-microbes model the top features include *Coprococcus eutactus*, *Ruminococcus gnavus*, *Fusobacterium nucleatum*, *Lachnospira sp*., and *Prevotella copri. Coprococcus eutactus* has a very high feature importance score and is the microbe with the highest association to RCC in our genes-and-microbes model (Table 5). *C. eutactus* has previously been associated with longer cancer progression-free survival, and was not found in the microbes-only model, which could hint at a genomic-microbial interaction between *C. eutactus* and CRC side [33]. *Ruminococcus gnavus, Ruminococcus sp.* and *Lachnospira* were previously identified as being important to CRC and associated with structural segregation of the mucosa [34]. *Fusobacterium nucleatum* was found to be important in the genes-and-microbes model, but was not discovered by the microbes-only model. *F. nucleatum* is one of the most commonly associated species with CRC, and it is believed to act as a pathobiont [35, 36]. It is also known to cause periodontal disease and is currently being explored as a biomarker for high-risk CRC. Given that *F. nucleatum* was only significant in the genes-and-microbes model, and that it is known to be only situationally pathogenic, this suggests this taxon may become pathogenic under specific gene co-activation [35, 36]. *Prevotella copri*, also identified uniquely by our genes-and-microbes model, has been shown to be significantly enriched in the gut microbiome of CRC patients compared with normal patients [37].

### Patterns in LCC

One recurring pattern in LCC is the expression of genes known to be associated with the prostate or prostate cancer. This is of interest given the heightened prevalence of LCC in men, and the left-sided colon’s close proximity to the prostate [38]. Prostate cancers and LCCs can be challenging to distinguish from biopsy samples, due to similarities in morphology and immunohistochemistry [39]. Genes that are of high importance in our RF models that are associated with both LCC and prostate cancer include *PRAC1*, *HOXB13*, *SPAG16* (Tables 3 and 5) [26]. PRAC1 has been previously associated with LCC as well as prostate cancer [21, 40]. HOXB13 has a protective effect against tumor proliferation in RCC [41], and a reduction in the expression of HOXB13 via hypermethylation of the DNMT3B-HOXB13-C-myc signaling axis has been associated with tumor proliferation and metastasis in RCC [41]. Our results indicated that HOXB13 is under-expressed in RCC relative to LCC, which adds evidence to the hypothesis that decreased HOXB13 expression is specifically associated with RCC [41]. Elevated HOXC6 has been linked to poor overall survival in LCC patients, but not RCC patients [41]. *MYOM3* was the top-ranking feature in our genes-and-microbes model and has a higher expression in LCC as determined using differential gene expression analysis. *MYOM3* has not been studied in CRC but it has been linked to clinical outcomes in renal and lung cancer [42, 43].

While our microbes-only model mostly identified microbes associated with RCC, *Akkermansia muciphila* was more common in LCC (Table 4). *A. muciniphila* degrades mucin in the gut, and has previously been shown to exacerbate colitis-associated CRC development in mice [44], and is associated with total pathological response in treatment of non-small cell lung cancer [45]. *Akkermansia* has been noted as one of three microbes most likely to have a causal association with differential CRC treatment effectiveness [10].

The genes-and-microbes model also identified microbes that for the most part were enriched in RCC (Table 5). Only two microbes in this model are present at higher levels in LCC, namely, *Verrucomicrobia bacterium* and *Fimbriiglobus ruber*. *Verrucomicrobia* has been studied as a biomarker for the early detection of CRC [46]. However, *Fimbriiglobus* is largely uncharacterized.

## Conclusions

Understanding microbial-genomic interactions may be important for informing treatment regimens in colorectal cancer. This study uses machine learning random forest (RF) models and differential gene expression (DE) to discover and associate genetic and microbial biomarkers with LCC and RCC. Three RF models with accuracy scores of 0.9, 0.7, and 0.87 were created and these yielded 15, 54 and 46 significantly important features. DE analysis was used to quantify changes in expression between CRC side. Our genes-and-microbes model identified microbes that did not appear in our microbes-only model, including *C. eutactus*, *F. nucleatum* and *P. copri*, and this may indicate that the random forest model is uncovering interactive effects between genes and microbes. RCC was most associated with the *HOX* family of genes, including *HOX*-associated lncRNA AC012531.25. LCC was highly associated with prostate cancer related genes, which is of interest as LCC is more common in men. The future of CRC research lies in personalized genomics, and the biomarkers identified by these three classification models may play an important role in the observed variability in clinicopathological and treatment outcomes of CRC patients.

## Electronic supplementary material

Below is the link to the electronic supplementary material.

Supplementary Material 1

## Data Availability

Raw Sequence Reads available at SRA Accession: PRJNA788974. Code is available from: https://github.com/tkolisnik/Kolisnik-Identifying-Important-CRC-Biomarkers-With-RF. The R package Rf2pval used in these analyses is available at: www.github.com/tkolisnik/Rf2pval. SciKit Learn can be imported from: https://scikit-learn.org/stable/modules/generated/sklearn.ensemble.RandomForestClassifier.html.
