# Supplementary material for: Identifying important microbial and genomic biomarkers for differentiating right- versus left-sided colorectal cancer using random forest models
Source: BMC Cancer. 2023 Jul 11;23:647. doi: 10.1186/s12885-023-10848-9 (PMC10337110; doi:10.1186/s12885-023-10848-9)
Supplement: Supplementary file 1 — Supplementary Material 1 [file 12885_2023_10848_MOESM1_ESM.docx]

## Supplementary Material


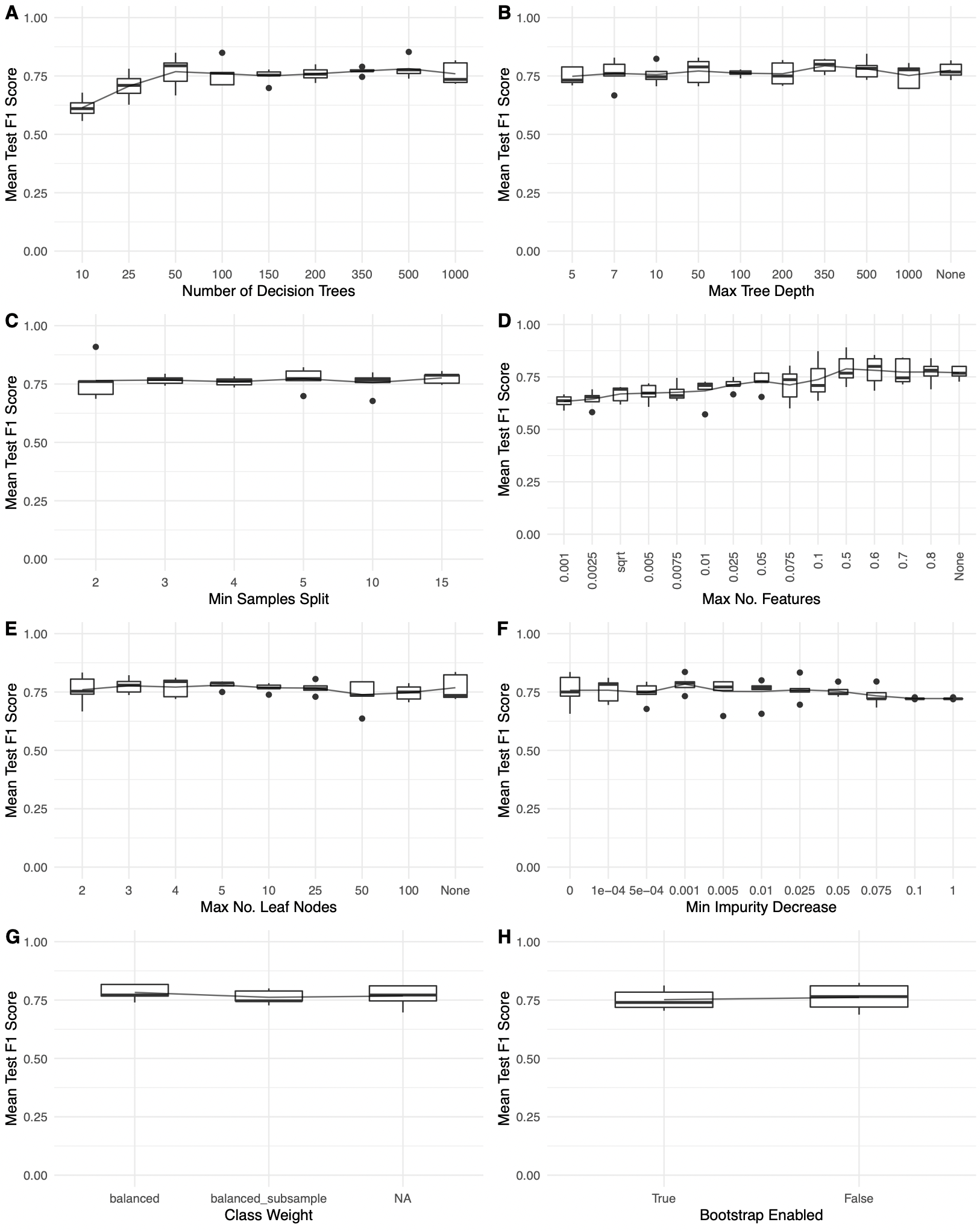


**Supplementary Fig. 1** Mean test F1 scores over 8 hyperparameters set to a varying range of intervals, used in building the RF genes-only model for purposes of narrowing down the hyperparameters searched during GridSearchCV to speed up computing time.


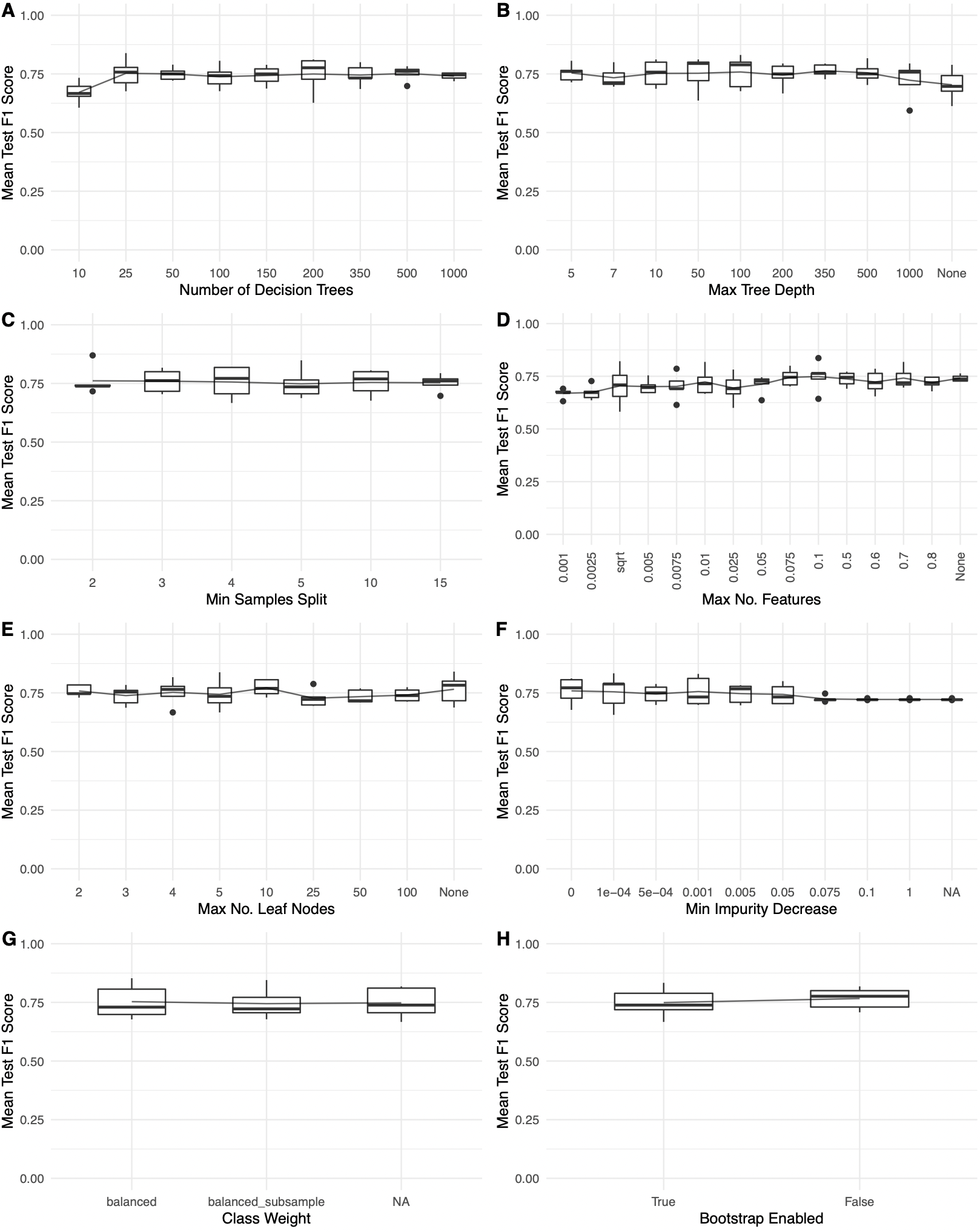


**Supplementary Fig. 2** Mean test F1 scores over 8 hyperparameters set to a varying range of intervals, used in building the RF microbial-only model for purposes of narrowing down the hyperparameters searched during GridSearchCV to speed up computing time.


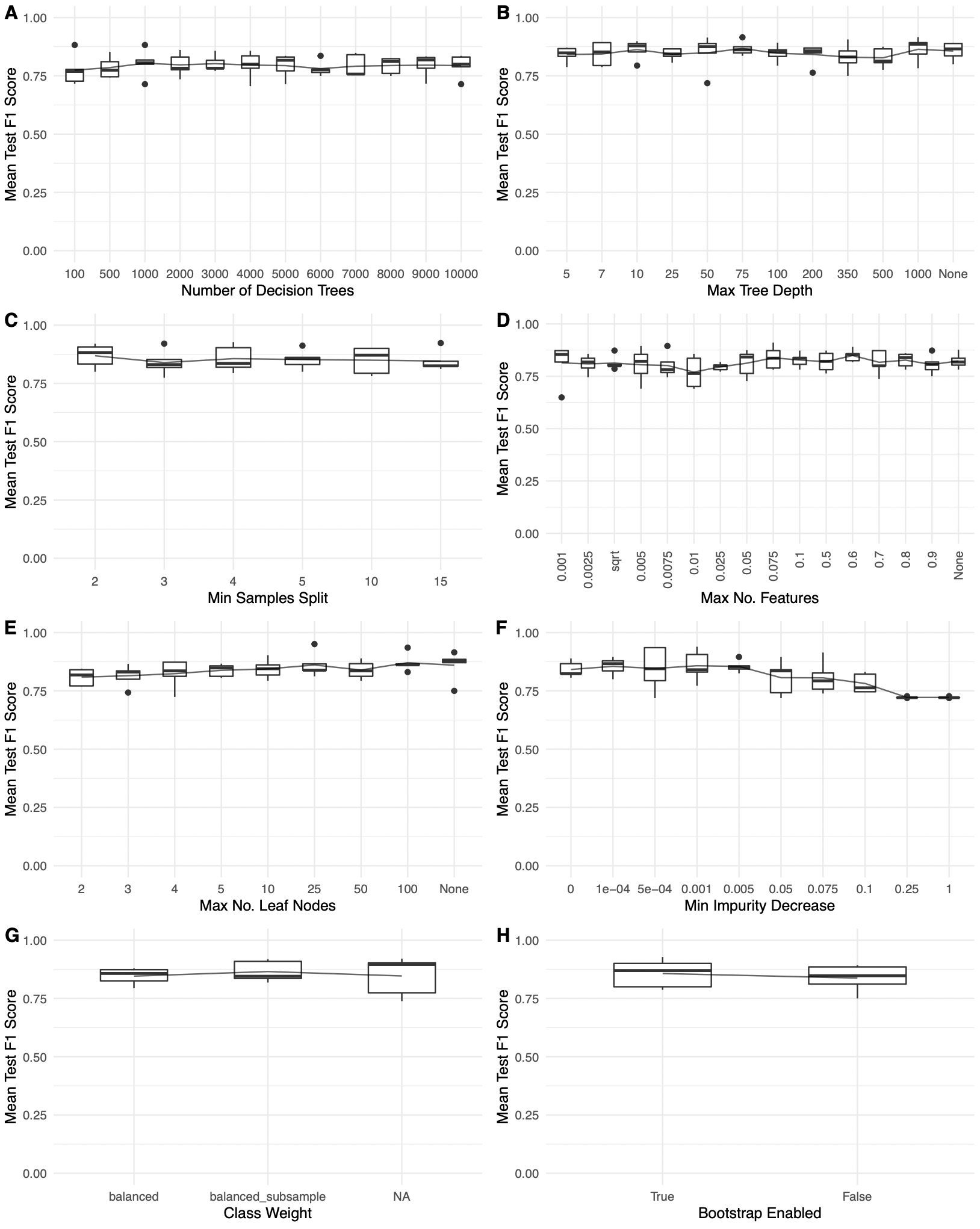


**Supplementary Fig. 3** Mean test F1 scores over eight hyperparameters set to a varying range of intervals, used in building the RF genes-and-microbes model for purposes of narrowing down the hyperparameters searched during GridSearchCV to speed up computing time.


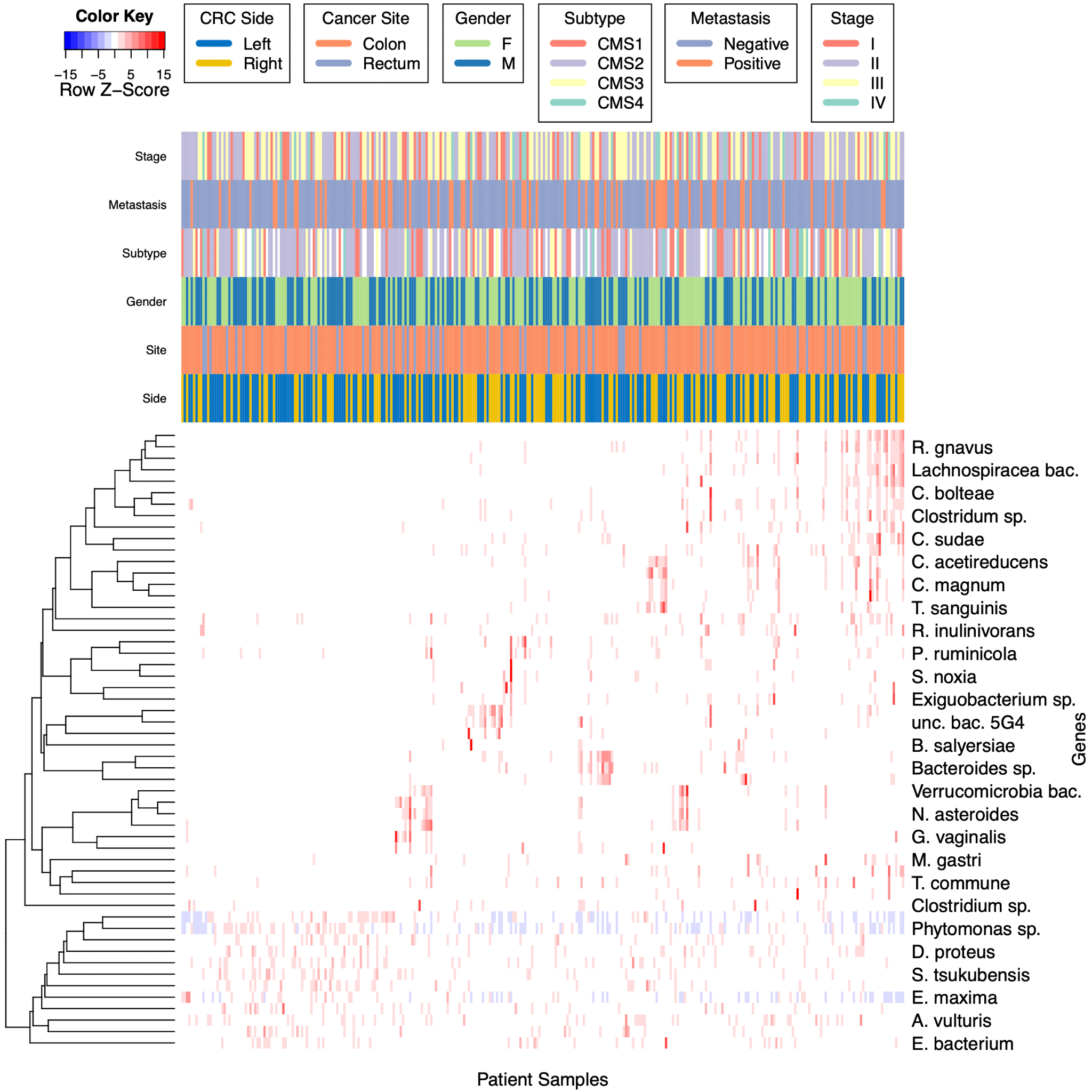


**Supplementary Fig. 4** A heatmap of scaled gene expression values of the top-scoring microbial features discovered by the microbes-only RF model and clinical characteristics. Hierarchical clustering of both genes and patients is via Pearson correlation, based on average linkage distance. The colors indicate row-scaled z-scores of TPM RNA-seq gene expression ratios.

###


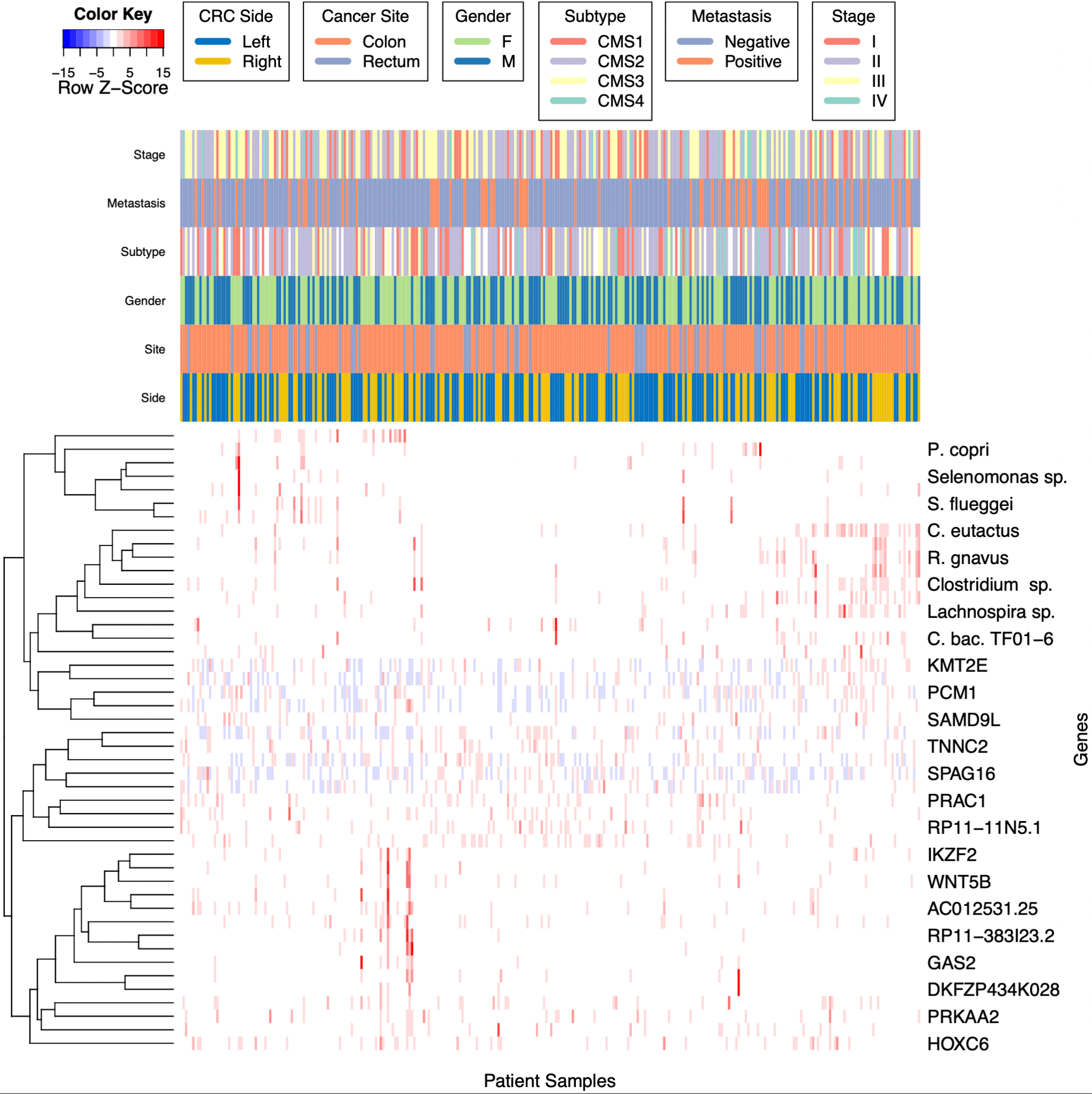
**Supplementary Fig. 5** A heatmap of scaled genomic and microbial expression values of the top-scoring features discovered by the genes-and-microbes RF model and clinical characteristics. Hierarchical clustering of both genes and patients is via Pearson correlation, based on average linkage distance. The colors indicate row-scaled z-scores of TPM RNA-seq gene expression ratios, as well as CPM normalized RNA-seq human unmapped microbial reads.

**Supplementary Table 1.** Full version of table 4. Top ranking features with p-values less than 0.05 and their importance scores discovered by our microbes-only model (Left). Side-paired differential expression (fold change) analysis results of CPM values for the same features (Right) Wilcoxon-rank sum test was used to calculate p-values and FDR (Benjamini & Hochberg).

| **Model Feature Importance Metrics** | | | | | **Differential Expression** | | | |
| --- | --- | --- | --- | --- | --- | --- | --- | --- |
| **Rank** | **Tax ID_Name** | **Importance Score** | **Log Importance Score** | **p-value** | **Log2 FC** | **p-value** | **FDR** | **Greater Expr. Side** |
| 1 | 33038_[Ruminococcus] gnavus | 0.025 | -3.69 | 0 | 2.07E+00 | 1.31E-15 | 3.54E-14 | Right |
| 2 | 76489_Clostridium acetireducens | 0.020 | -3.91 | 0 | 1.88E+00 | 1.69E-13 | 1.52E-12 | Right |
| 3 | 1701326_uncultured bacterium 5G4 | 0.018 | -4.04 | 0 | 1.73E+00 | 6.55E-11 | 2.52E-10 | Right |
| 4 | 397291_Lachnospiraceae bacterium A4 | 0.014 | -4.27 | 0 | 2.30E+00 | 5.29E-16 | 2.85E-14 | Right |
| 5 | 2293240_Ruminococcus sp. TF10-6 | 0.013 | -4.34 | 0 | 2.41E+00 | 2.86E-15 | 5.14E-14 | Right |
| 6 | 239935_Akkermansia muciniphila | 0.011 | -4.51 | 0 | -5.30E-01 | 2.27E-05 | 3.96E-05 | Left |
| 7 | 1531_[Clostridium] clostridioforme | 0.011 | -4.55 | 0 | 1.47E+00 | 1.73E-12 | 1.03E-11 | Right |
| 8 | 936381_Selenomonas sp. CM52 | 0.010 | -4.61 | 0 | 4.03E+00 | 8.68E-11 | 3.13E-10 | Right |
| 9 | 46228_Ruminococcus lactaris | 0.009 | -4.76 | 0 | 1.86E+00 | 2.96E-12 | 1.45E-11 | Right |
| 10 | 43064_Trichococcus pasteurii | 0.007 | -4.97 | 0 | 2.18E+00 | 1.67E-08 | 4.10E-08 | Right |
| 11 | 1262831_Clostridium sp. CAG:678 | 0.007 | -5.01 | 0 | 1.3 | 0.0322 | 0.0464 | Right |
| 12 | 1824_Nocardia asteroides | 0.007 | -5.02 | 0 | -0.19 | 0.00112 | 0.00172 | Left |
| 13 | 208479_[Clostridium] bolteae | 0.007 | -5.03 | 0 | 1.76E+00 | 2.18E-13 | 1.68E-12 | Right |
| 14 | 2026799_Verrucomicrobia bacterium | 0.006 | -5.14 | 0.01 | -0.17 | 2.02E-06 | 3.76E-06 | Left |
| 15 | 1262706_Azospirillum sp. CAG:260 | 0.006 | -5.14 | 0 | 1.15 | 1.86E-09 | 5.57E-09 | Right |
| 16 | 360807_Roseburia inulinivorans | 0.006 | -5.15 | 0 | 1.9 | 1.49E-12 | 1.01E-11 | Right |
| 17 | 83915_Distigma proteus | 0.006 | -5.17 | 0 | 0.29 | 0.109 | 0.137 | Right |
| 18 | 1328_Streptococcus anginosus | 0.006 | -5.19 | 0 | -0.36 | 0.106 | 0.136 | Left |
| 19 | 747377_Clostridium sp. DMHC 10 | 0.006 | -5.20 | 0 | 1.99 | 2.12E-09 | 6.04E-09 | Right |
| 20 | 91626_Mucor ambiguus | 0.006 | -5.20 | 0 | 0.83 | 1.19E-09 | 3.78E-09 | Right |
| 21 | 1741_Thermodesulfobacterium commune | 0.005 | -5.21 | 0 | -0.73 | 0.54 | 0.583 | Left |
| 22 | 88431_Dorea longicatena | 0.005 | -5.23 | 0 | 2.01 | 2.12E-14 | 2.86E-13 | Right |
| 23 | 2702_Gardnerella vaginalis | 0.005 | -5.25 | 0 | -0.49 | 0.624 | 0.661 | Left |
| 24 | 33954_Clostridium magnum | 0.005 | -5.26 | 0 | 2.33 | 2.69E-08 | 6.32E-08 | Right |
| 25 | 291644_Bacteroides salyersiae | 0.005 | -5.27 | 0 | 2.17 | 0.00102 | 0.00161 | Right |
| 26 | 105841_Anaerostipes caccae | 0.005 | -5.27 | 0 | 1.67 | 1.90E-12 | 1.03E-11 | Right |
| 27 | 839_Prevotella ruminicola | 0.005 | -5.27 | 0 | 0.95 | 0.172 | 0.197 | Right |
| 28 | 134013_Phytomonas sp. isolate Hart1 | 0.005 | -5.28 | 0 | -0.29 | 0.0379 | 0.0525 | Left |
| 29 | 1262910_Oscillibacter sp. CAG:155 | 0.005 | -5.29 | 0 | -0.26 | 0.833 | 0.849 | Left |
| 30 | 83656_Streptomyces tsukubensis | 0.005 | -5.31 | 0 | -0.68 | 0.000729 | 0.00119 | Left |
| 31 | 135083_Selenomonas noxia | 0.005 | -5.35 | 0 | 3.44 | 8.73E-06 | 1.57E-05 | Right |
| 32 | 162156_uncultured Bacteroides sp. | 0.005 | -5.35 | 0 | 0.39 | 1.87E-07 | 3.60E-07 | Right |
| 33 | 39486_Dorea formicigenerans | 0.005 | -5.35 | 0 | 1.23 | 4.61E-08 | 9.96E-08 | Right |
| 34 | 872327_Lactobacillus pasteurii | 0.005 | -5.35 | 0 | 1.21 | 1.31E-08 | 3.54E-08 | Right |
| 35 | 1736298_Exiguobacterium sp. Leaf196 | 0.005 | -5.38 | 0 | 2.89 | 3.47E-12 | 1.56E-11 | Right |
| 36 | 310300_Bacteroides pyogenes | 0.004 | -5.41 | 0 | 1.76 | 3.29E-08 | 7.41E-08 | Right |
| 37 | 260710_Olavius algarvensis spirochete endosymbiont | 0.004 | -5.42 | 0 | 1.8 | 1.66E-08 | 4.10E-08 | Right |
| 38 | 1522369_Cyanophora sudae | 0.004 | -5.46 | 0 | 1.61 | 1.19E-13 | 1.28E-12 | Right |
| 39 | 857335_Candidatus Dactylopiibacterium carminicum | 0.004 | -5.46 | 0 | 0.15 | 0.14 | 0.168 | Right |
| 40 | 261299_Intestinibacter bartlettii | 0.004 | -5.48 | 0 | 2 | 2.23E-11 | 9.24E-11 | Right |
| 41 | 564198_Mycolicibacterium bacteremicum | 0.004 | -5.50 | 0 | -0.82 | 0.0132 | 0.0198 | Left |
| 42 | 411484_Clostridium sp. SS2/1 | 0.004 | -5.51 | 0 | 1.99 | 1.44E-10 | 4.87E-10 | Right |
| 43 | 1777_Mycobacterium gastri | 0.004 | -5.57 | 0.01 | 0.17 | 0.418 | 0.471 | Right |
| 44 | 1857645_Actinomyces vulturis | 0.004 | -5.58 | 0.01 | 0.47 | 0.161 | 0.189 | Right |
| 45 | 11768_Feline leukemia virus | 0.004 | -5.58 | 0.01 | -0.49 | 6.37E-05 | 0.000107 | Left |
| 46 | 1736483_Microbacterium sp. Root180 | 0.004 | -5.58 | 0.01 | -0.7 | 0.129 | 0.159 | Left |
| 47 | 1768115_bacterium F082 | 0.004 | -5.58 | 0.01 | 1.44 | 9.65E-08 | 1.93E-07 | Right |
| 48 | 877415_Erysipelotrichaceae bacterium NK3D112 | 0.004 | -5.60 | 0.01 | 0.64 | 0.0327 | 0.0464 | Right |
| 49 | 1632858_Massilibacterium senegalense | 0.004 | -5.61 | 0.01 | -0.09 | 0.444 | 0.489 | Left |
| 50 | 5804_Eimeria maxima | 0.004 | -5.61 | 0.01 | -0.19 | 0.0681 | 0.0919 | Left |
| 51 | 154288_Turicibacter sanguinis | 0.004 | -5.62 | 0.01 | 1.9 | 5.00E-08 | 1.04E-07 | Right |
| 52 | 490622_Trichoderma arundinaceum | 0.004 | -5.63 | 0.01 | -0.04 | 0.791 | 0.821 | Left |
| 53 | 653385_Actinomyces sp. oral taxon 849 | 0.004 | -5.63 | 0.01 | -0.05 | 0.938 | 0.938 | Left |
| 54 | 467085_Candidatus Symbiothrix dinenymphae | 0.004 | -5.65 | 0.01 | -1.95 | 0.0923 | 0.122 | Left |

**Supplementary Table 2.** Full version of table 5. Top ranking features with p-values less than 0.05 and their importance scores discovered by our genes-and-microbes model (Left). Side-paired differential expression (fold change) analysis results of TPM and CPM values for the same features (Right) Wilcoxon-rank sum test was used to calculate p-values and FDR (Benjamini & Hochberg).

| **Model Feature Importance Metrics** | | | | | **Differential Expression** | | | |
| --- | --- | --- | --- | --- | --- | --- | --- | --- |
| **Rank** | **ENSG ID_Gene/Tax ID_Name** | **Importance Score** | **Log Importance Score** | **p-value** | **Log2 FC** | **p-value** | **FDR** | **Associated Side** |
| 1 | ENSG00000142661_MYOM3 | 0.037 | -3.29 | 0 | -0.62 | 1.36E-08 | 2.71E-08 | Left |
| 2 | ENSG00000198353_HOXC4 | 0.033 | -3.40 | 0 | 1.88 | 2.11E-15 | 3.23E-14 | Right |
| 3 | 33043_Coprococcus eutactus | 0.027 | -3.62 | 0 | 2.08 | 2.00E-14 | 1.54E-13 | Right |
| 4 | ENSG00000159182_PRAC1 | 0.024 | -3.72 | 0 | -2.86 | 5.08E-21 | 2.34E-19 | Left |
| 5 | ENSG00000260597_AC012531.25 | 0.020 | -3.90 | 0 | 1.19 | 4.26E-12 | 2.17E-11 | Right |
| 6 | 33038_[Ruminococcus] gnavus | 0.016 | -4.13 | 0 | 2.07 | 1.31E-15 | 3.02E-14 | Right |
| 7 | ENSG00000184719_RNLS | 0.011 | -4.50 | 0.01 | -0.89 | 2.85E-10 | 9.37E-10 | Left |
| 8 | ENSG00000197757_HOXC6 | 0.011 | -4.54 | 0 | 1.28 | 5.02E-12 | 2.31E-11 | Right |
| 9 | ENSG00000144451_SPAG16 | 0.010 | -4.61 | 0 | -0.64 | 9.00E-09 | 1.97E-08 | Left |
| 10 | 851_Fusobacterium nucleatum | 0.010 | -4.62 | 0 | 1.67 | 2.73E-06 | 3.49E-06 | Right |
| 11 | ENSG00000273374_RP11-383I23.2 | 0.010 | -4.62 | 0 | -1.02 | 2.86E-08 | 5.47E-08 | Left |
| 12 | 446043_uncultured Lachnospira sp. | 0.010 | -4.63 | 0 | 1.46 | 5.97E-09 | 1.37E-08 | Right |
| 13 | 165179_Prevotella copri | 0.009 | -4.66 | 0 | 1.6 | 1.50E-09 | 3.85E-09 | Right |
| 14 | 154288_Turicibacter sanguinis | 0.009 | -4.67 | 0 | 1.9 | 5.00E-08 | 8.51E-08 | Right |
| 15 | 59620_uncultured Clostridium sp. | 0.009 | -4.67 | 0 | 1.09 | 6.86E-12 | 2.87E-11 | Right |
| 16 | ENSG00000101470_TNNC2 | 0.009 | -4.69 | 0 | -1.29 | 3.34E-10 | 1.02E-09 | Left |
| 17 | 936381_Selenomonas sp. CM52 | 0.009 | -4.69 | 0 | 4.03 | 8.68E-11 | 3.07E-10 | Right |
| 18 | ENSG00000162409_PRKAA2 | 0.009 | -4.69 | 0 | -1.2 | 3.90E-10 | 1.12E-09 | Left |
| 19 | ENSG00000124915_DKFZP434K028 | 0.009 | -4.70 | 0 | 0.91 | 1.67E-05 | 1.97E-05 | Right |
| 20 | 712991_Lachnospiraceae bacterium oral taxon 500 | 0.009 | -4.71 | 0 | 2.33 | 9.20E-07 | 1.28E-06 | Right |
| 21 | ENSG00000101076_HNF4A | 0.009 | -4.72 | 0 | -0.48 | 4.73E-08 | 8.37E-08 | Left |
| 22 | ENSG00000177409_SAMD9L | 0.009 | -4.72 | 0 | 1.01 | 7.55E-08 | 1.20E-07 | Right |
| 23 | 2293240_Ruminococcus sp. TF10-6 | 0.009 | -4.72 | 0 | 2.41 | 2.86E-15 | 3.28E-14 | Right |
| 24 | ENSG00000146386_ABRACL | 0.009 | -4.72 | 0 | -0.29 | 1.71E-04 | 0.000192 | Left |
| 25 | 2305245_Clostridiaceae bacterium TF01-6 | 0.009 | -4.74 | 0 | 1.79 | 1.96E-14 | 1.54E-13 | Right |
| 26 | ENSG00000111186_WNT5B | 0.009 | -4.75 | 0 | -0.53 | 2.65E-04 | 0.00029 | Left |
| 27 | ENSG00000188610_FAM72B | 0.009 | -4.75 | 0 | 0.67 | 2.58E-06 | 3.39E-06 | Right |
| 28 | ENSG00000250829_RP11-11N5.1 | 0.009 | -4.75 | 0 | -3.79 | 1.14E-08 | 2.39E-08 | Left |
| 29 | ENSG00000126218_F10 | 0.009 | -4.75 | 0 | -0.55 | 4.46E-10 | 1.21E-09 | Left |
| 30 | 28133_Prevotella nigrescens | 0.009 | -4.75 | 0 | 1.27 | 5.70E-08 | 9.37E-08 | Right |
| 31 | ENSG00000103534_TMC5 | 0.009 | -4.76 | 0 | 0.29 | 7.29E-03 | 0.0078 | Right |
| 32 | 113574_Hyphomicrobium sp. GJ21 | 0.009 | -4.77 | 0 | 2.24 | 2.10E-09 | 5.09E-09 | Right |
| 33 | ENSG00000078674_PCM1 | 0.008 | -4.78 | 0 | 0.39 | 3.38E-08 | 6.22E-08 | Right |
| 34 | 2026799_Verrucomicrobia bacterium | 0.008 | -4.79 | 0 | -0.17 | 2.02E-06 | 2.73E-06 | Left |
| 35 | 105841_Anaerostipes caccae | 0.008 | -4.80 | 0 | 1.67 | 1.90E-12 | 1.09E-11 | Right |
| 36 | 2320113_bacterium 1xD42-87 | 0.008 | -4.80 | 0 | 1.4 | 2.73E-07 | 4.05E-07 | Right |
| 37 | ENSG00000005483_KMT2E | 0.008 | -4.81 | 0 | 0.15 | 2.08E-02 | 0.0213 | Right |
| 38 | ENSG00000197217_ENTPD4 | 0.008 | -4.81 | 0 | 0.3 | 9.00E-07 | 1.28E-06 | Right |
| 39 | ENSG00000030419_IKZF2 | 0.008 | -4.81 | 0 | 0.48 | 1.36E-05 | 1.64E-05 | Right |
| 40 | 135080_Selenomonas flueggei | 0.008 | -4.81 | 0 | 3.21 | 9.96E-13 | 6.54E-12 | Right |
| 41 | ENSG00000166173_LARP6 | 0.008 | -4.84 | 0 | -0.8 | 1.21E-05 | 1.51E-05 | Left |
| 42 | ENSG00000267506_RP11-13K12.1 | 0.008 | -4.85 | 0 | -1.79 | 1.16E-11 | 4.43E-11 | Left |
| 43 | ENSG00000148935_GAS2 | 0.008 | -4.87 | 0 | 0.05 | 2.34E-07 | 3.59E-07 | Right |
| 44 | ENSG00000026559_KCNG1 | 0.008 | -4.87 | 0 | -0.22 | 5.56E-02 | 0.0556 | Left |
| 45 | 1908690_Fimbriiglobus ruber | 0.007 | -4.90 | 0.04 | -0.46 | 2.04E-02 | 0.0213 | Left |
| 46 | ENSG00000144891_AGTR1 | 0.007 | -4.91 | 0.03 | -1.83 | 4.72E-05 | 5.42E-05 | Left |

#

**Supplementary Table 3 - Patient Demographics & Cancer Characteristics Stratified by Cancer Side.** An alternative version of Table 1, stratified by LCC and RCC patient groups.

| **Characteristic** | **LCC Patients** | **RCC Patients** |
| --- | --- | --- |
| **Patients enrolled - no (%)** | 172 (56) | 136 (44) |
| **Median Age - year (range)** | 72.7 (28.7, 89.8) | 75.3 (36.9, 91.5) |
| **Sex** |  |  |
| **Female - no (%)** | 79 (26) | 84 (27) |
| **Male - no (%)** | 93 (30) | 52 (17) |
| **Metastasis** |  |  |
| **Positive - no (%)** | 44 (14) | 26 (8) |
| **Negative - no (%)** | 128 (42) | 110 (36) |
| **Cancer Stage** |  |  |
| **T1 - no (%)** | 34 (11) | 19 (6) |
| **T2 - no (%)** | 63 (20.5) | 65 (21) |
| **T3 - no (%)** | 63 (20.5) | 42 (14) |
| **T4 - no (%)** | 12 (4) | 10 (3) |
| **Nodal Status** |  |  |
| **Positive - no (%)** | 98 (32) | 87 (28) |
| **Negative - no (%)** | 74 (24) | 49 (16) |

**Supplementary Code 1.**

Python script for creating the random forest classifier and obtaining the scoring metrics reported in the results for the genes-only classifier. Random forest hyperparameters can also be found within this code, for reproducibility purposes.

A Genes-Only Random Forest Classifier for CRC Anatomical Side.py

#!/usr/bin/env python

# coding: utf-8

#Created by: Tyler Kolisnik

#March 9, 2023

# Description:

#This code is for evaluating and finalizing a random forest model after using grid search cv to find the best parameters

#Includes scoring and validation

# Import required packages and functions

import sklearn

import numpy as np

import pandas as pd

import rpy2.robjects as robjects

import pickle

import seaborn as sns

import matplotlib.pyplot as plt

from scipy import interpolate

from sklearn.ensemble import RandomForestClassifier

from sklearn.model_selection import train_test_split

from sklearn import metrics

from rpy2.robjects import pandas2ri

from sklearn.metrics import confusion_matrix, make_scorer

from sklearn.metrics import classification_report

from sklearn.metrics import precision_recall_curve

from sklearn.metrics import roc_curve

from matplotlib import pyplot

# Increase output plot resolution

plt.rcParams['figure.dpi'] = 300

plt.rcParams['savefig.dpi'] = 300

sns.set(rc={"figure.dpi":300, 'savefig.dpi':300})

sns.set_context('notebook')

sns.set_style("whitegrid")

# Import the training set data (278 samples)

pandas2ri.activate()

readRDS = robjects.r['readRDS']

ml_data=readRDS('/Volumes/FryShareNVME/Preprocessing_Workflow/2022-05-23 preprocessing workflow_SIDE_SPLIT/output_data/2022-03-25-ML-VALUES-discovery-set-side-278samples.rds')

tpmdata=ml_data[0]

tpmdata2=tpmdata.transpose()

targetdata=np.ravel(ml_data[1]).astype(int)

targetlabels=np.ravel(ml_data[2])

# Create the classifier from the best parameter set found in the grid search CV

rforest_finalparamset = RandomForestClassifier(

bootstrap=True,

class_weight=None,

criterion='gini',

max_depth=5,

max_features=0.1,

max_leaf_nodes=4,

min_impurity_decrease=0.0,

min_samples_leaf=1,

min_samples_split=2,

min_weight_fraction_leaf=0.0,

n_estimators=25,

n_jobs=2,

oob_score=True,

random_state=2,

verbose=0,

warm_start=False

)

# Split the dataset into training and testing

#Test Size = 0.25 instructs the model to fit on 75% of the data and test on 25%

#Stratify ensures an even distribution of samples

#Fixing random state allows for reproducibility of results

X_train, X_test, y_train, y_test = train_test_split(tpmdata2, targetdata,

test_size=0.25,random_state=1,

stratify=targetdata,shuffle=True)

# Fit (train) the classifier to our dataset

#alternatively import this from a file if you have already saved the model (.pkl)

#rforest_finalparamset.fit(X_train,y_train)

# Load the model from disk

#filename = '/Volumes/FryShareNVME/2022-03-25-ML-Results-Side/2022-03-26-ML-paramset-analysis/crc-side-random-forest-model.pkl'

filename = '/Volumes/FryShareNVME/2022-03-25-ML-Results-Side/2022-03-27-finalized-paper-data/genes-rf-exploration/crc-side-random-forest-model.pkl'

rforest_finalparamset = pickle.load(open(filename, 'rb'))

# Save the model to disk

#filename = '/Volumes/FryShareNVME/2022-03-25-ML-Results-Side/2022-03-26-ML-paramset-analysis/crc-side-random-forest-model.pkl'

#pickle.dump(rforest_finalparamset, open(filename, 'wb'))

# Make predictions from the training data

rfpredictions=rforest_finalparamset.predict(X_test)

print("Model Accuracy:",metrics.accuracy_score(y_test,rfpredictions))

# Threshold hyperparameter optimization

#The default threshold for a binary variable is 0.5

#Checking the threshold allows for the optimization of the sensitivity and specificity

#and a correction for imbalanced datasets

#If a new threshold is set then you must nolonger use .predict, and must use .predict_proba

# Generate prediction probabilities (necessary for AUROC/AUPRC and thresholds)

yhat = rforest_finalparamset.predict_proba(X_test)

# keep probabilities for the positive outcome only

yhat = yhat[:, 1]

# calculate roc curves

fpr, tpr, thresholds = roc_curve(y_test, yhat)

# calculate the g-mean for each threshold

gmeans = np.sqrt(tpr * (1-fpr))

# locate the index of the largest g-mean

ix = np.argmax(gmeans)

print('Best Threshold=%f, G-Mean=%.3f' % (thresholds[ix], gmeans[ix]))

# plot the roc curve for the model

pyplot.plot([0,1], [0,1], linestyle='--', label='No Skill')

#t=np.linspace(0, 2*np.pi,1000)

pyplot.plot(fpr, tpr, marker='.', label='Logistic')

#pyplot.plot(t, np.interp(t,fpr, tpr), marker='.', label='Cubic Spline')

pyplot.scatter(fpr[ix], tpr[ix], marker='o', color='black', label='Best')

# axis labels

pyplot.xlabel('False Positive Rate')

pyplot.ylabel('True Positive Rate')

pyplot.legend()

# show the plot

pyplot.show()

#With Code Adapted From: https://machinelearningmastery.com/threshold-moving-for-imbalanced-classification/

# See how a threshold adjustment affects the training set

#I ultimately chose to just keep the 0.5 threshold as the scores are close enough

#and it is not of utmost importance in this model to prioritize

#precision(specificity) or recall(sensitivity) over the other

threshold = 0.539077

thresholdpredictions = rforest_finalparamset.predict_proba(X_test)

discpreds2 = thresholdpredictions[:, 1]

test_predictions=rforest_finalparamset.predict(X_test)

preds = [1 if thresholdpredictions[i][1]> threshold else 0 for i in range(len(thresholdpredictions))]

print("0.539077 Threshold:")

pd.DataFrame(classification_report(y_test,preds,output_dict=True)).T

print("0.5 Threshold:")

pd.DataFrame(classification_report(y_test,test_predictions,output_dict=True)).T

# Print Scoring Metrics for Training (Model Discovery) Set

print("Accuracy:",metrics.accuracy_score(y_test,rfpredictions))

print("Out-of-Bag Score:",rforest_finalparamset.oob_score_)

print("F1 Score:",metrics.f1_score(y_test,rfpredictions))

print("ROC AUC Score:",metrics.roc_auc_score(y_test,rfpredictions))

print("Recall Score:",metrics.recall_score(y_test,rfpredictions))

print("Precision Score:",metrics.precision_score(y_test,rfpredictions))

confmat = confusion_matrix(y_test,rfpredictions)

fp = confmat[0][1]

tn = confmat[0][0]

tp = confmat[1][1]

fn = confmat[1][0]

tprate=tp/(tp+fn)

fprate=fp/(fp+tn)

print("False Positives:",fp)

print("False Negatives:",fn)

print("True Positives:",tp)

print("True Negatives:",tn)

print("True Positive Rate:",tprate)

print("False Positive Rate:",fprate)

print(classification_report(y_test,rfpredictions))

# Generate AUROC/AUPRC scores and create functions for plotting curves

val_auprc = sklearn.metrics.average_precision_score(y_test,rfpredictions,pos_label=1)

val_auroc = sklearn.metrics.roc_auc_score(y_test,rfpredictions)

aurocscore="AUROC="+str(round(val_auroc,2))

auprcscore="AUPRC="+str(round(val_auprc,2))

print(auprcscore)

print(aurocscore)

def plot_auroc_curve(y_test,rfpredictions,aurocscore):

data_fpr, data_tpr, data_thresholds = sklearn.metrics.roc_curve(y_test,rfpredictions)

data_fpr_tpr = pd.DataFrame({'fpr':data_fpr, 'tpr':data_tpr})

p = sns.lineplot(data=data_fpr_tpr, x='fpr', y='tpr',ci=None)

p.set(xlabel='False Positive Rate', ylabel='True Positive Rate')

p.plot([0, 1], [0, 1], color='black', ls='--')

p.text(0.2,0.5,aurocscore)

def plot_auprc_curve(y_test,rfpredictions,auprcscore):

data_prcsn, data_rcll, data_thrshlds = sklearn.metrics.precision_recall_curve(y_test,rfpredictions)

data_prcsn_rcll = pd.DataFrame({'prcsn':data_prcsn, 'rcll':data_rcll})

p = sns.lineplot(data=data_prcsn_rcll, x='rcll', y='prcsn',ci=None)

p.set(xlabel='Recall', ylabel='Precision')

p.plot([0, 1], [1, 0], color='black', ls='--')

p.text(0.2,0.5,auprcscore)

# Plot AUROC Curve

plot_auroc_curve(y_test,discpreds2,aurocscore)

# Save to file

plt.savefig("/Volumes/FryShareNVME/2022-03-25-ML-Results-Side/2022-03-27-finalized-paper-data/genes-rf-exploration/plots-for-paper/AUROC-training-set-v2genesonly.pdf")

# Plot AUPRC Curve

plot_auprc_curve(y_test,discpreds2,auprcscore)

plt.show()

# Save to file

plt.savefig("/Volumes/FryShareNVME/2022-03-25-ML-Results-Side/2022-03-27-finalized-paper-data/genes-rf-exploration/plots-for-paper/AUPRC-training-set-v2genesonly.pdf")

# Import independent validation set (30 samples the model has never seen before)

pandas2ri.activate()

readRDS = robjects.r['readRDS']

val_data=readRDS('/Volumes/FryShareNVME/Preprocessing_Workflow/2022-05-23 preprocessing workflow_SIDE_SPLIT/output_data/2022-03-25-ML-VALUES-validation-set-side-30samples.rds')

valdata=val_data[0]

valdata2=valdata.transpose()

valtargetdata=np.ravel(val_data[1]).astype(int)

# Run the model on the validation set

runmodelonvaldata=rforest_finalparamset.predict(valdata2)

# Generate prediction probabilities (necessary for AUROC/AUPRC and thresholds)

val_prob_preds = rforest_finalparamset.predict_proba(valdata2)

# Take only the positive values

val_preds = val_prob_preds[:, 1]

# Print Scoring Metrics for Validation Set

print("Accuracy:",metrics.accuracy_score(runmodelonvaldata, valtargetdata))

print("F1 Score:",metrics.f1_score(runmodelonvaldata, valtargetdata))

print("ROC AUC Score:",metrics.roc_auc_score(runmodelonvaldata, valtargetdata))

print("Recall Score:",metrics.recall_score(runmodelonvaldata, valtargetdata))

print("Precision Score:",metrics.precision_score(runmodelonvaldata, valtargetdata))

confmat = confusion_matrix(runmodelonvaldata,valtargetdata)

fp = confmat[0][1]

tn = confmat[0][0]

tp = confmat[1][1]

fn = confmat[1][0]

tprate=tp/(tp+fn)

fprate=fp/(fp+tn)

print("False Positives:",fp)

print("False Negatives:",fn)

print("True Positives:",tp)

print("True Negatives:",tn)

print("True Positive Rate:",tprate)

print("True Negative Rate:",fprate)

print(classification_report(runmodelonvaldata, valtargetdata))

# Generate validation set AUROC/AUPRC scores

val_auprc = sklearn.metrics.average_precision_score(runmodelonvaldata, valtargetdata,pos_label=1)

val_auroc = sklearn.metrics.roc_auc_score(runmodelonvaldata, valtargetdata)

aurocscore="AUROC="+str(round(val_auroc,2))

auprcscore="AUPRC="+str(round(val_auprc,2))

print(auprcscore)

print(aurocscore)

# Plot AUROC Curve

auroccurve=plot_auroc_curve(valtargetdata,val_preds,aurocscore)

# Save to file

plt.savefig("/Volumes/FryShareNVME/2022-03-25-ML-Results-Side/2022-03-27-finalized-paper-data/genes-rf-exploration/plots-for-paper/AUROC-validation-set-v2-genesonly.pdf")

# Plot AUPRC Curve

plot_auprc_curve(valtargetdata,val_preds,auprcscore)

# Save to file

plt.savefig("/Volumes/FryShareNVME/2022-03-25-ML-Results-Side/2022-03-27-finalized-paper-data/genes-rf-exploration/plots-for-paper/AUPRC-validation-set-v2-genesonly.pdf")

# See how a threshold adjustment affects the validation set (as a post-measure of performance, not for threshold optimization)

#I ultimately chose to just keep the 0.5 threshold as the scores are close enough

#and it is not of utmost importance in this model to prioritize

#precision(specificity) or recall(sensitivity) over the other

threshold = 0.539077 # define threshold here

thresholdpredictions = rforest_finalparamset.predict_proba(valdata2)

valpredictions=rforest_finalparamset.predict(valdata2)

preds = [1 if thresholdpredictions[i][1]> threshold else 0 for i in range(len(thresholdpredictions))]

# Print Classification Report for New Threshold

print("0.539077 Threshold:")

pd.DataFrame(classification_report(valtargetdata,preds,output_dict=True)).T

# Print Classification Report for 0.5 Threshold (Default)

print("0.5 Threshold:")

pd.DataFrame(classification_report(valtargetdata,valpredictions,output_dict=True)).T

# Save the feature importance scores of the random forest model mapped to the features (genes)

ftnames=rforest_finalparamset.feature_names_in_

ftimportances=rforest_finalparamset.feature_importances_

df=pd.DataFrame({ 'feature_names':ftnames,'feature_importances':ftimportances,})

# Save to file

df.to_csv("/Volumes/FryShareNVME/2022-03-25-ML-Results-Side/2022-03-26-ML-paramset-analysis/2022-03-27-crc-rf-final-feature-importance-scores.csv")

**Supplementary Code 2.**

Python script for creating the random forest classifier and obtaining the scoring metrics reported in the results for the microbial-only classifier. Random forest hyperparameters can also be found within this code, for reproducibility purposes.

A Microbial-Only Random Forest Classifier for CRC Anatomical Side.py

#!/usr/bin/env python

# coding: utf-8

#Created by: Tyler Kolisnik

#March 9, 2023

# Description:

#This code is for evaluating and finalizing a random forest model after using grid search cv to find the best parameters

#Includes scoring and validation

# Import required packages and functions

import sklearn

import numpy as np

import pandas as pd

import rpy2.robjects as robjects

import pickle

import seaborn as sns

import matplotlib.pyplot as plt

from sklearn.ensemble import RandomForestClassifier

from sklearn.model_selection import train_test_split

from sklearn import metrics

from rpy2.robjects import pandas2ri

from sklearn.metrics import confusion_matrix, make_scorer

from sklearn.metrics import classification_report

from sklearn.metrics import roc_curve

from matplotlib import pyplot

# Increase output plot resolution

plt.rcParams['figure.dpi'] = 300

plt.rcParams['savefig.dpi'] = 300

sns.set(rc={"figure.dpi":300, 'savefig.dpi':300})

sns.set_context('notebook')

sns.set_style("whitegrid")

# Import the training set data (278 samples)

pandas2ri.activate()

readRDS = robjects.r['readRDS']

ml_data=readRDS('/Volumes/FryShareNVME/2022-03-25-ML-Results-Side/2022-03-27-finalized-paper-data/microbial-rf-exploration /2022-05-29-microbial-only preprocessing workflow_SIDE/output_data/2022-03-29-CPM-MicrobialONLY-discovery-set-side-278samples.rds')

tpmdata=ml_data[0]

tpmdata2=tpmdata.transpose()

targetdata=np.ravel(ml_data[1]).astype(int)

targetlabels=np.ravel(ml_data[2])

# Create the classifier from the best parameter set found in the grid search CV

rforest_finalparamset = RandomForestClassifier(

bootstrap=True,

class_weight=None,

criterion='gini',

max_depth=5,

max_features=0.1,

max_leaf_nodes=10,

min_impurity_decrease=0.0,

min_samples_leaf=1,

min_samples_split=2,

min_weight_fraction_leaf=0.0,

n_estimators=100,

n_jobs=2,

oob_score=True,

random_state=2,

verbose=0,

warm_start=False

)

# Split the dataset into training and testing

#Test Size = 0.25 instructs the model to fit on 75% of the data and test on 25%

#Stratify ensures an even distribution of samples

#Fixing random state allows for reproducibility of results

X_train, X_test, y_train, y_test = train_test_split(tpmdata2, targetdata,

test_size=0.25,random_state=1,

stratify=targetdata,shuffle=True)

# Fit (train) the classifier to our dataset

#alternatively import this from a file if you have already saved the model (.pkl)

rforest_finalparamset.fit(X_train,y_train)

# Load the model from disk

#filename = '/Volumes/FryShareNVME/2022-03-25-ML-Results-Side/2022-03-26-ML-paramset-analysis/crc-side-random-forest-model.pkl'

#rforest_finalparamset = pickle.load(open(filename, 'rb'))

# Save the model to disk

#filename = '/Volumes/FryShareNVME/2022-03-25-ML-Results-Side/2022-03-26-ML-paramset-analysis/crc-side-random-forest-model.pkl'

#pickle.dump(rforest_finalparamset, open(filename, 'wb'))

# Make predictions from the training data

rfpredictions=rforest_finalparamset.predict(X_test)

print("Model Accuracy:",metrics.accuracy_score(y_test,rfpredictions))

# Threshold hyperparameter optimization

#The default threshold for a binary variable is 0.5

#Checking the threshold allows for the optimization of the sensitivity and specificity

#and a correction for imbalanced datasets

#If a new threshold is set then you must nolonger use .predict, and must use .predict_proba

# Generate prediction probabilities (necessary for AUROC/AUPRC and thresholds)

yhat = rforest_finalparamset.predict_proba(X_test)

# keep probabilities for the positive outcome only

yhat = yhat[:, 1]

# calculate roc curves

fpr, tpr, thresholds = roc_curve(y_test, yhat)

# calculate the g-mean for each threshold

gmeans = np.sqrt(tpr * (1-fpr))

# locate the index of the largest g-mean

ix = np.argmax(gmeans)

print('Best Threshold=%f, G-Mean=%.3f' % (thresholds[ix], gmeans[ix]))

# plot the roc curve for the model

pyplot.plot([0,1], [0,1], linestyle='--', label='No Skill')

pyplot.plot(fpr, tpr, marker='.', label='Logistic')

pyplot.scatter(fpr[ix], tpr[ix], marker='o', color='black', label='Best')

# axis labels

pyplot.xlabel('False Positive Rate')

pyplot.ylabel('True Positive Rate')

pyplot.legend()

# show the plot

pyplot.show()

#With Code Adapted From: https://machinelearningmastery.com/threshold-moving-for-imbalanced-classification/

# See how a threshold adjustment affects the training set

#I ultimately chose to just keep the 0.5 threshold as the scores are close enough

#and it is not of utmost importance in this model to prioritize

#precision(specificity) or recall(sensitivity) over the other

threshold = 0.530549

thresholdpredictions = rforest_finalparamset.predict_proba(X_test)

discpreds2 = thresholdpredictions[:, 1]

test_predictions=rforest_finalparamset.predict(X_test)

preds = [1 if thresholdpredictions[i][1]> threshold else 0 for i in range(len(thresholdpredictions))]

print("0.530549 Threshold:")

pd.DataFrame(classification_report(y_test,preds,output_dict=True)).T

print("0.5 Threshold:")

pd.DataFrame(classification_report(y_test,test_predictions,output_dict=True)).T

# Print Scoring Metrics for Training (Model Discovery) Set

print("Accuracy:",metrics.accuracy_score(y_test,rfpredictions))

print("Out-of-Bag Score:",rforest_finalparamset.oob_score_)

print("F1 Score:",metrics.f1_score(y_test,rfpredictions))

print("ROC AUC Score:",metrics.roc_auc_score(y_test,rfpredictions))

print("Recall Score:",metrics.recall_score(y_test,rfpredictions))

print("Precision Score:",metrics.precision_score(y_test,rfpredictions))

confmat = confusion_matrix(y_test,rfpredictions)

fp = confmat[0][1]

tn = confmat[0][0]

tp = confmat[1][1]

fn = confmat[1][0]

tprate=tp/(tp+fn)

fprate=fp/(fp+tn)

print("False Positives:",fp)

print("False Negatives:",fn)

print("True Positives:",tp)

print("True Negatives:",tn)

print("True Positive Rate:",tprate)

print("True Negative Rate:",fprate)

print(classification_report(y_test,rfpredictions))

# Generate AUROC/AUPRC scores and create functions for plotting curves

val_auprc = sklearn.metrics.average_precision_score(y_test,rfpredictions,pos_label=1)

val_auroc = sklearn.metrics.roc_auc_score(y_test,rfpredictions)

aurocscore="AUROC="+str(round(val_auroc,2))

auprcscore="AUPRC="+str(round(val_auprc,2))

print(auprcscore)

print(aurocscore)

def plot_auroc_curve(y_test,rfpredictions,aurocscore):

data_fpr, data_tpr, data_thresholds = sklearn.metrics.roc_curve(y_test,rfpredictions)

data_fpr_tpr = pd.DataFrame({'fpr':data_fpr, 'tpr':data_tpr})

p = sns.lineplot(data=data_fpr_tpr, x='fpr', y='tpr',ci=None)

p.set(xlabel='False Positive Rate', ylabel='True Positive Rate')

p.plot([0, 1], [0, 1], color='black', ls='--')

p.text(0.2,0.5,aurocscore)

def plot_auprc_curve(y_test,rfpredictions,auprcscore):

data_prcsn, data_rcll, data_thrshlds = sklearn.metrics.precision_recall_curve(y_test,rfpredictions)

data_prcsn_rcll = pd.DataFrame({'prcsn':data_prcsn, 'rcll':data_rcll})

p = sns.lineplot(data=data_prcsn_rcll, x='rcll', y='prcsn',ci=None)

p.set(xlabel='Recall', ylabel='Precision')

p.plot([0, 1], [1, 0], color='black', ls='--')

p.text(0.2,0.5,auprcscore)

# Plot AUROC Curve

plot_auroc_curve(y_test,discpreds2,aurocscore)

# Save to file

plt.savefig("/Volumes/FryShareNVME/2022-03-25-ML-Results-Side/2022-03-27-finalized-paper-data/microbial-rf-exploration /microbialonly-AUROC-training-set.pdf")

# Plot AUPRC Curve

plot_auprc_curve(y_test,discpreds2,auprcscore)

# Save to file

plt.savefig("/Volumes/FryShareNVME/2022-03-25-ML-Results-Side/2022-03-27-finalized-paper-data/microbial-rf-exploration /microbialonly-AUPRC-training-set.pdf")

# Import independent validation set (30 samples the model has never seen before)

pandas2ri.activate()

readRDS = robjects.r['readRDS']

val_data=readRDS('/Volumes/FryShareNVME/2022-03-25-ML-Results-Side/2022-03-27-finalized-paper-data/microbial-rf-exploration /2022-05-29-microbial-only preprocessing workflow_SIDE/output_data/2022-03-29-CPM-MicrobialONLY-validation-set-side-30samples.rds')

valdata=val_data[0]

valdata2=valdata.transpose()

valtargetdata=np.ravel(val_data[1]).astype(int)

# Run the model on the validation set

runmodelonvaldata=rforest_finalparamset.predict(valdata2)

# Generate prediction probabilities (necessary for AUROC/AUPRC and thresholds)

val_prob_preds = rforest_finalparamset.predict_proba(valdata2)

# Take only the positive values

val_preds = val_prob_preds[:, 1]

# Print Scoring Metrics for Validation Set

print("Accuracy:",metrics.accuracy_score(runmodelonvaldata, valtargetdata))

print("F1 Score:",metrics.f1_score(runmodelonvaldata, valtargetdata))

print("ROC AUC Score:",metrics.roc_auc_score(runmodelonvaldata, valtargetdata))

print("Recall Score:",metrics.recall_score(runmodelonvaldata, valtargetdata))

print("Precision Score:",metrics.precision_score(runmodelonvaldata, valtargetdata))

confmat = confusion_matrix(runmodelonvaldata,valtargetdata)

fp = confmat[0][1]

tn = confmat[0][0]

tp = confmat[1][1]

fn = confmat[1][0]

tprate=tp/(tp+fn)

fprate=fp/(fp+tn)

print("False Positives:",fp)

print("False Negatives:",fn)

print("True Positives:",tp)

print("True Negatives:",tn)

print("True Positive Rate:",tprate)

print("True Negative Rate:",fprate)

print(classification_report(runmodelonvaldata, valtargetdata))

# Generate validation set AUROC/AUPRC scores

val_auprc = sklearn.metrics.average_precision_score(runmodelonvaldata, valtargetdata,pos_label=1)

val_auroc = sklearn.metrics.roc_auc_score(runmodelonvaldata, valtargetdata)

aurocscore="AUROC="+str(round(val_auroc,2))

auprcscore="AUPRC="+str(round(val_auprc,2))

print(auprcscore)

print(aurocscore)

# Plot AUROC Curve

auroccurve=plot_auroc_curve(valtargetdata,val_preds,aurocscore)

# Save to file

plt.savefig("/Volumes/FryShareNVME/2022-03-25-ML-Results-Side/2022-03-27-finalized-paper-data/microbial-rf-exploration /microbialonly-AUROC-validation-set.pdf")

# Plot AUPRC Curve

plot_auprc_curve(valtargetdata,val_preds,auprcscore)

# Save to file

plt.savefig("/Volumes/FryShareNVME/2022-03-25-ML-Results-Side/2022-03-27-finalized-paper-data/microbial-rf-exploration /microbialonly-AUPRC-validation-set.pdf")

# See how a threshold adjustment affects the validation set (as a post-measure of performance, not for threshold optimization)

#I ultimately chose to just keep the 0.5 threshold as the scores are close enough

#and it is not of utmost importance in this model to prioritize

#precision(specificity) or recall(sensitivity) over the other

threshold = 0.539077 # define threshold here

thresholdpredictions = rforest_finalparamset.predict_proba(valdata2)

valpredictions=rforest_finalparamset.predict(valdata2)

preds = [1 if thresholdpredictions[i][1]> threshold else 0 for i in range(len(thresholdpredictions))]

# Print Classification Report for New Threshold

print("0.530549 Threshold:")

pd.DataFrame(classification_report(valtargetdata,preds,output_dict=True)).T

# Print Classification Report for 0.5 Threshold (Default)

print("0.5 Threshold:")

pd.DataFrame(classification_report(valtargetdata,valpredictions,output_dict=True)).T

# Save the feature importance scores of the random forest model mapped to the features (genes)

ftnames=rforest_finalparamset.feature_names_in_

ftimportances=rforest_finalparamset.feature_importances_

df=pd.DataFrame({ 'feature_names':ftnames,'feature_importances':ftimportances,})

# Save to file

df.to_csv("/Volumes/FryShareNVME/2022-03-25-ML-Results-Side/2022-03-27-finalized-paper-data/microbial-rf-exploration /2022-04-05-microbialonly-crc-rf-final-feature-importance-scores.csv")

**Supplementary Code 3.**

Python script for creating the random forest classifier and obtaining the scoring metrics reported in the results for the genes-and-microbes classifier. Random forest hyperparameters can also be found within this code, for reproducibility purposes.

A Genes-And-Microbes Random Forest Classifier for CRC Anatomical Side.py

#!/usr/bin/env python

# coding: utf-8

#Created by: Tyler Kolisnik

#March 9, 2023

# Description:

#This code is for evaluating and finalizing a random forest model after using grid search cv to find the best parameters

#Includes scoring and validation

# Import required packages and functions

import sklearn

import numpy as np

import pandas as pd

import rpy2.robjects as robjects

import pickle

import seaborn as sns

import matplotlib.pyplot as plt

from sklearn.ensemble import RandomForestClassifier

from sklearn.model_selection import train_test_split

from sklearn import metrics

from rpy2.robjects import pandas2ri

from sklearn.metrics import confusion_matrix, make_scorer

from sklearn.metrics import classification_report

from sklearn.metrics import roc_curve

from matplotlib import pyplot

# Increase output plot resolution

plt.rcParams['figure.dpi'] = 300

plt.rcParams['savefig.dpi'] = 300

sns.set(rc={"figure.dpi":300, 'savefig.dpi':300})

sns.set_context('notebook')

sns.set_style("whitegrid")

# Import the training set data (278 samples)

pandas2ri.activate()

readRDS = robjects.r['readRDS']

ml_data=readRDS('/Volumes/FryShareNVME/2022-03-25-ML-Results-Side/2022-03-27-finalized-paper-data/microbial-plusallgenes-exploration/2022-05-29-microbial-plus-all-genes-preprocessing workflow_SIDE/output_data/2022-03-29-CPM-Microbial-PLUS-GENES-discovery-set-side-278samples.rds')

tpmdata=ml_data[0]

tpmdata2=tpmdata.transpose()

targetdata=np.ravel(ml_data[1]).astype(int)

targetlabels=np.ravel(ml_data[2])

# Create the classifier from the best parameter set found in the grid search CV

rforest_finalparamset = RandomForestClassifier(

bootstrap=True,

class_weight=None,

criterion='gini',

max_depth=10,

max_features=0.025,

max_leaf_nodes=4,

min_impurity_decrease=0.0,

min_samples_leaf=1,

min_samples_split=2,

min_weight_fraction_leaf=0.0,

n_estimators=50,

n_jobs=2,

oob_score=True,

random_state=2,

verbose=0,

warm_start=False

)

# Split the dataset into training and testing

#Test Size = 0.25 instructs the model to fit on 75% of the data and test on 25%

#Stratify ensures an even distribution of samples

#Fixing random state allows for reproducibility of results

X_train, X_test, y_train, y_test = train_test_split(tpmdata2, targetdata,

test_size=0.25,random_state=3,

stratify=targetdata,shuffle=True)

# Fit (train) the classifier to our dataset

#alternatively import this from a file if you have already saved the model (.pkl)

rforest_finalparamset.fit(X_train,y_train)

# Load the model from disk

#filename = '/Volumes/FryShareNVME/2022-03-25-ML-Results-Side/2022-03-27-finalized-paper-data/microbial-plusallgenes-exploration/2022-04-03-genesplusbacteria-crc-side-random-forest-model.pkl'

#rforest_finalparamset = pickle.load(open(filename, 'rb'))

#rforest_finalparamset.score(X_test, Y_test)

# Make predictions from the training data

rfpredictions=rforest_finalparamset.predict(X_test)

print("Model Accuracy:",metrics.accuracy_score(y_test,rfpredictions))

# Threshold hyperparameter optimization

#The default threshold for a binary variable is 0.5

#Checking the threshold allows for the optimization of the sensitivity and specificity

#and a correction for imbalanced datasets

#If a new threshold is set then you must nolonger use .predict, and must use .predict_proba

# Generate prediction probabilities (necessary for AUROC/AUPRC and thresholds)

yhat = rforest_finalparamset.predict_proba(X_test)

# keep probabilities for the positive outcome only

yhat = yhat[:, 1]

# calculate roc curves

fpr, tpr, thresholds = roc_curve(y_test, yhat)

# calculate the g-mean for each threshold

gmeans = np.sqrt(tpr * (1-fpr))

# locate the index of the largest g-mean

ix = np.argmax(gmeans)

print('Best Threshold=%f, G-Mean=%.3f' % (thresholds[ix], gmeans[ix]))

# plot the roc curve for the model

pyplot.plot([0,1], [0,1], linestyle='--', label='No Skill')

pyplot.plot(fpr, tpr, marker='.', label='Logistic')

pyplot.scatter(fpr[ix], tpr[ix], marker='o', color='black', label='Best')

# axis labels

pyplot.xlabel('False Positive Rate')

pyplot.ylabel('True Positive Rate')

pyplot.legend()

# show the plot

pyplot.show()

#With Code Adapted From: https://machinelearningmastery.com/threshold-moving-for-imbalanced-classification/

# See how a threshold adjustment affects the training set

#I ultimately chose to just keep the 0.5 threshold as the scores are close enough

#and it is not of utmost importance in this model to prioritize

#precision(specificity) or recall(sensitivity) over the other

threshold = 0.554755

thresholdpredictions = rforest_finalparamset.predict_proba(X_test)

discpreds2 = thresholdpredictions[:, 1]

test_predictions=rforest_finalparamset.predict(X_test)

preds = [1 if thresholdpredictions[i][1]> threshold else 0 for i in range(len(thresholdpredictions))]

print("0.554755 Threshold:")

pd.DataFrame(classification_report(y_test,preds,output_dict=True)).T

print("0.5 Threshold:")

pd.DataFrame(classification_report(y_test,test_predictions,output_dict=True)).T

# Print Scoring Metrics for Training (Model Discovery) Set

print("Accuracy:",metrics.accuracy_score(y_test,rfpredictions))

print("Out-of-Bag Score:",rforest_finalparamset.oob_score_)

print("F1 Score:",metrics.f1_score(y_test,rfpredictions))

print("ROC AUC Score:",metrics.roc_auc_score(y_test,rfpredictions))

print("Recall Score:",metrics.recall_score(y_test,rfpredictions))

print("Precision Score:",metrics.precision_score(y_test,rfpredictions))

confmat = confusion_matrix(y_test,rfpredictions)

fp = confmat[0][1]

tn = confmat[0][0]

tp = confmat[1][1]

fn = confmat[1][0]

tprate=tp/(tp+fn)

fprate=fp/(fp+tn)

print("False Positives:",fp)

print("False Negatives:",fn)

print("True Positives:",tp)

print("True Negatives:",tn)

print("True Positive Rate:",tprate)

print("True Negative Rate:",fprate)

print(classification_report(y_test,rfpredictions))

# Generate AUROC/AUPRC scores and create functions for plotting curves

val_auprc = sklearn.metrics.average_precision_score(y_test,rfpredictions,pos_label=1)

val_auroc = sklearn.metrics.roc_auc_score(y_test,rfpredictions)

aurocscore="AUROC="+str(round(val_auroc,2))

auprcscore="AUPRC="+str(round(val_auprc,2))

print(auprcscore)

print(aurocscore)

def plot_auroc_curve(y_test,rfpredictions,aurocscore):

data_fpr, data_tpr, data_thresholds = sklearn.metrics.roc_curve(y_test,rfpredictions)

data_fpr_tpr = pd.DataFrame({'fpr':data_fpr, 'tpr':data_tpr})

p = sns.lineplot(data=data_fpr_tpr, x='fpr', y='tpr',ci=None)

p.set(xlabel='False Positive Rate', ylabel='True Positive Rate')

p.plot([0, 1], [0, 1], color='black', ls='--')

p.text(0.2,0.5,aurocscore)

def plot_auprc_curve(y_test,rfpredictions,auprcscore):

data_prcsn, data_rcll, data_thrshlds = sklearn.metrics.precision_recall_curve(y_test,rfpredictions)

data_prcsn_rcll = pd.DataFrame({'prcsn':data_prcsn, 'rcll':data_rcll})

p = sns.lineplot(data=data_prcsn_rcll, x='rcll', y='prcsn',ci=None)

p.set(xlabel='Recall', ylabel='Precision')

p.plot([0, 1], [1, 0], color='black', ls='--')

p.text(0.2,0.5,auprcscore)

# Plot AUROC Curve

plot_auroc_curve(y_test,discpreds2,aurocscore)

plot_auroc_curve

# Save to file

plt.savefig("/Volumes/FryShareNVME/2022-03-25-ML-Results-Side/2022-03-27-finalized-paper-data/microbial-plusallgenes-exploration/bacteriaplusgenes-v2-AUROC-training-set.pdf")

# Plot AUPRC Curve

plot_auprc_curve(y_test,discpreds2,auprcscore)

# Save to file

plt.savefig("/Volumes/FryShareNVME/2022-03-25-ML-Results-Side/2022-03-27-finalized-paper-data/microbial-plusallgenes-exploration/bacteriaplusgenes-v2-AUPRC-training-set.pdf")

# Import independent validation set (30 samples the model has never seen before)

pandas2ri.activate()

readRDS = robjects.r['readRDS']

val_data=readRDS('/Volumes/FryShareNVME/2022-03-25-ML-Results-Side/2022-03-27-finalized-paper-data/microbial-plusallgenes-exploration/2022-05-29-microbial-plus-all-genes-preprocessing workflow_SIDE/output_data/2022-03-29-CPM-Microbial-PLUS-GENES-validation-set-side-30samples.rds')

valdata=val_data[0]

valdata2=valdata.transpose()

valtargetdata=np.ravel(val_data[1]).astype(int)

# Run the model on the validation set

runmodelonvaldata=rforest_finalparamset.predict(valdata2)

# Generate prediction probabilities (necessary for AUROC/AUPRC and thresholds)

val_prob_preds = rforest_finalparamset.predict_proba(valdata2)

# Take only the positive values

val_preds = val_prob_preds[:, 1]

# Print Scoring Metrics for Validation Set

print("Accuracy:",metrics.accuracy_score(runmodelonvaldata, valtargetdata))

print("F1 Score:",metrics.f1_score(runmodelonvaldata, valtargetdata))

print("ROC AUC Score:",metrics.roc_auc_score(runmodelonvaldata, valtargetdata))

print("Recall Score:",metrics.recall_score(runmodelonvaldata, valtargetdata))

print("Precision Score:",metrics.precision_score(runmodelonvaldata, valtargetdata))

confmat = confusion_matrix(runmodelonvaldata,valtargetdata)

fp = confmat[0][1]

tn = confmat[0][0]

tp = confmat[1][1]

fn = confmat[1][0]

tprate=tp/(tp+fn)

fprate=fp/(fp+tn)

print("False Positives:",fp)

print("False Negatives:",fn)

print("True Positives:",tp)

print("True Negatives:",tn)

print("True Positive Rate:",tprate)

print("True Negative Rate:",fprate)

print(classification_report(runmodelonvaldata, valtargetdata))

# Generate validation set AUROC/AUPRC scores

val_auprc = sklearn.metrics.average_precision_score(runmodelonvaldata, valtargetdata,pos_label=1)

val_auroc = sklearn.metrics.roc_auc_score(runmodelonvaldata, valtargetdata)

aurocscore="AUROC="+str(round(val_auroc,2))

auprcscore="AUPRC="+str(round(val_auprc,2))

print(auprcscore)

print(aurocscore)

# Plot AUROC Curve

auroccurve=plot_auroc_curve(valtargetdata,val_preds,aurocscore)

auroccurve

# Save to file

plt.savefig("/Volumes/FryShareNVME/2022-03-25-ML-Results-Side/2022-03-27-finalized-paper-data/microbial-plusallgenes-exploration/bacteriaplusgenes-v2-AUROC-validation-set.pdf")

# Plot AUPRC Curve

plot_auprc_curve(valtargetdata,val_preds,auprcscore)

# Save to file

plt.savefig("/Volumes/FryShareNVME/2022-03-25-ML-Results-Side/2022-03-27-finalized-paper-data/microbial-plusallgenes-exploration/bacteriaplusgenes-v2-AUPRC-validation-set.pdf")

# See how a threshold adjustment affects the validation set (as a post-measure of performance, not for threshold optimization)

#I ultimately chose to just keep the 0.5 threshold as the scores are close enough

#and it is not of utmost importance in this model to prioritize

#precision(specificity) or recall(sensitivity) over the other

threshold = 0.554755 # define threshold here

thresholdpredictions = rforest_finalparamset.predict_proba(valdata2)

valpredictions=rforest_finalparamset.predict(valdata2)

preds = [1 if thresholdpredictions[i][1]> threshold else 0 for i in range(len(thresholdpredictions))]

# Print Classification Report for New Threshold

print("0.570738 Threshold:")

pd.DataFrame(classification_report(valtargetdata,preds,output_dict=True)).T

# Print Classification Report for 0.5 Threshold (Default)

print("0.5 Threshold:")

pd.DataFrame(classification_report(valtargetdata,valpredictions,output_dict=True)).T

# Save the feature importance scores of the random forest model mapped to the features (genes)

ftnames=rforest_finalparamset.feature_names_in_

ftimportances=rforest_finalparamset.feature_importances_

df=pd.DataFrame({ 'feature_names':ftnames,'feature_importances':ftimportances,})

# Save to file

df.to_csv("/Volumes/FryShareNVME/2022-03-25-ML-Results-Side/2022-03-27-finalized-paper-data/microbial-plusallgenes-exploration/2022-04-05-bacteriaplusgenes-v2-crc-rf-final-feature-importance-scores.csv")

# Save the model to disk

filename = '/Volumes/FryShareNVME/2022-03-25-ML-Results-Side/2022-03-27-finalized-paper-data/microbial-plusallgenes-exploration/2022-04-03-microbialplusallgenes-v2-crc-side-random-forest-model.pkl'

pickle.dump(rforest_finalparamset, open(filename, 'wb'))
